# ASCL2-mediated macrophage-myofibroblast transition generates immunosuppressive CAF_7 in NSCLC bone metastases

**DOI:** 10.1186/s13046-026-03735-1

**Published:** 2026-05-18

**Authors:** Jinfeng Wang, Jin Qian, Xiujia Yang, Chongquan Huang, Tiantian Wei, Jielong Zhou, Guoqing Zhong, Zhenhai Zhang, Yu Zhang

**Affiliations:** 1 Department of Orthopedics, Guangdong Provincial People’s Hospital, Guangdong Academy of Medical Sciences, Southern Medical University, Guangzhou, 510080 China; 2 Guangdong Engineering Technology Research Center of Functional Repair of Bone Defects and Biomaterials, Guangzhou, 510080 China; 3 Academy of Orthopedics, Guangdong Provincial Key Laboratory of Bone and Joint Degeneration Diseases, The Third Affiliated Hospital of Southern Medical University, Guangzhou, Guangdong Province 510630 China; 4 Center for Precision Medicine, Medical Research Institute, Guangdong Provincial People’s Hospital (Guangdong Academy of Medical Sciences), Southern Medical University, Guangzhou, 510080 China; 5 Guangdong-Hong Kong Joint Laboratory on Immunological and Genetic Kidney Diseases, Guangdong Provincial People’s Hospital (Guangdong Academy of Medical Sciences), Southern Medical University, Guangzhou, 510080 China; 6 Department of Bioinformatics, School of Basic Medical Sciences, Southern Medical University, Guangzhou, 510515 China; 7 Key Laboratory of Mental Health of the Ministry of Education, Guangdong-Hong Kong-Macao Greater Bay Area Center for Brain Science and Brain-Inspired Intelligence, Southern Medical University, Guangzhou, 510515 China; 8 Department of Bone and Soft Tissue Cancer, The Affiliated Cancer Hospital of Zhengzhou University & Henan Cancer Hospital, Dongming Road 127, Zhengzhou, 450008 China

**Keywords:** Non-small cell lung cancer (NSCLC), Bone metastases, Cancer-associated fibroblasts (CAF), Macrophage-myofibroblast transition (MMT), ASCL2, Immunosuppression, Tumor‐associated macrophages (TAM), Il6ra/IL6R

## Abstract

**Background:**

NSCLC frequently metastasizes to bone, where the microenvironment becomes immunosuppressive, limiting immunotherapy efficacy. Tumor-associated macrophages (TAMs) and cancer-associated fibroblasts (CAFs) contribute to immune evasion, but CAF subset origins and roles in bone metastases remain unclear. We investigated whether TAMs undergo macrophage-to-myofibroblast transition (MMT) to generate an immunosuppressive CAF subpopulation in bone lesions, driven by ASCL2.

**Methods:**

We integrated single-cell RNA sequencing of primary lung tumors and bone metastases with pseudotime and regulon analyses to resolve CAF heterogeneity and nominate MMT regulators. Findings were validated by immunofluorescence and flow cytometry in patient tissues and a murine bone-metastasis model. In vitro, TGF-β1-induced MMT with ASCL2 knockdown/rescue and T-cell co-culture assays were used to assess CAF_7-like induction and immunosuppressive function. In vivo, macrophage depletion/reconstitution, donor and endogenous tracing, and macrophage-directed ASCL2 or Il6ra knockdown were performed in a mouse bone-metastasis model to assess CAF_7-like abundance, tumor burden, and T-cell states; ASCL2 inhibition was further evaluated with PD-1 blockade for tumor control and survival.

**Results:**

Seven CAF subsets were identified, including a bone metastasis–enriched subset (CAF_7) that co-expressed macrophage, MHC-II, and stromal markers. Trajectory analyses, together with the observation that LLC-conditioned medium induced a CAF_7-like shift in bone marrow–derived macrophages in vitro and that both exogenous GFP-labeled BMDMs and endogenous macrophage-lineage-traced cells acquired CAF_7-like features in the bone metastatic microenvironment, supported TAM-to-CAF_7-like transition via MMT. CAF_7 accumulation correlated with increased Treg infiltration, CD8⁺ T cell exhaustion, and poorer survival. Regulon analysis highlighted ASCL2 as a CAF_7-associated regulator; ASCL2 increased in vitro during MMT, and in vivo ASCL2 protein and ASCL2⁺ CAF_7-like cells were enriched in bone-metastasis lesions versus sham marrow or subcutaneous tumors. ASCL2 knockdown in macrophages blocked MMT, reducing CAF_7-like cells and associated Treg and exhausted CD8⁺ T cells, and suppressing tumor growth. Mechanistically, ASCL2 directly transactivated Il6ra, and macrophage Il6ra knockdown phenocopied ASCL2 silencing in vivo. Notably, ASCL2 silencing synergized with anti–PD-1 therapy, improving tumor control and extending survival.

**Conclusions:**

In NSCLC bone metastases, ASCL2-driven MMT converts TAMs into immunosuppressive CAF_7 that promotes immune escape. Targeting ASCL2 disrupts this transition, restores anti-tumor immunity, and may improve immunotherapy efficacy.

**Graphical Abstract:**

**Figure:**
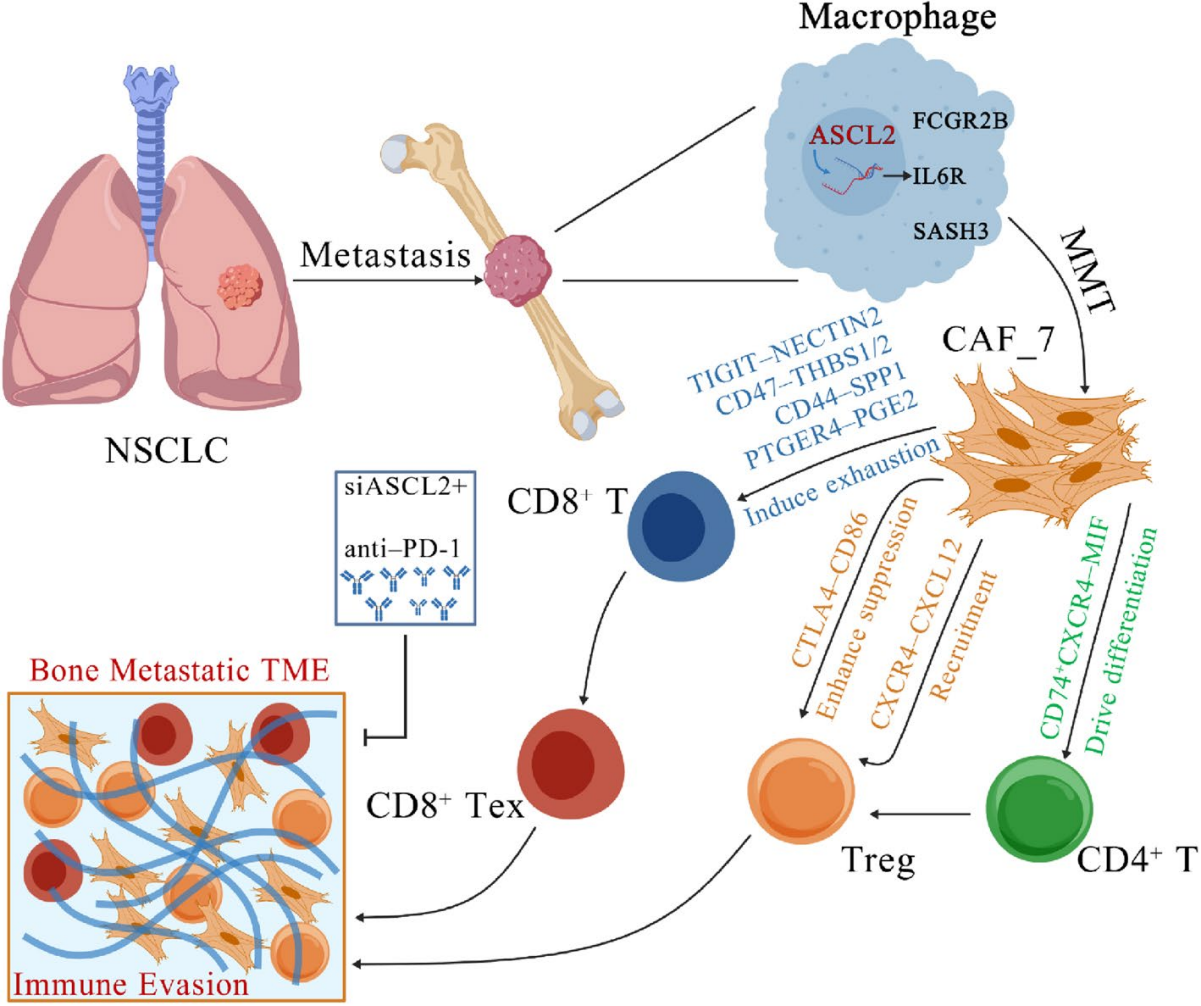
In NSCLC bone metastases, tumor-associated macrophages undergo ASCL2-driven macrophage–myofibroblast transition (MMT) to generate an immunosuppressive CAF subset (CAF_7). CAF_7 promotes Treg enrichment and CD8⁺ T-cell exhaustion. Targeting ASCL2 disrupts CAF_7 formation and enhances the antitumor activity of PD-1 blockade

**Supplementary Information:**

The online version contains supplementary material available at 10.1186/s13046-026-03735-1.

## Introduction

Non-small cell lung cancer (NSCLC) frequently metastasizes to bone, occurring in approximately 30–40% of advanced cases and markedly worsening patient prognosis [1]. Once tumor cells colonize the bone, the metastatic tumor microenvironment (TME) becomes highly conducive to immune evasion [2]. Interactions among tumor cells, bone-resorbing osteoclasts, osteoblasts, and immune cells in the bone marrow niche drive the release of immunosuppressive factors such as transforming growth factor-β (TGF-β), and promote the accumulation of regulatory T cells (Tregs) that dampen anti-tumor immunity. This immunosuppressive milieu—compounded by myeloid-derived suppressor cells (MDSCs) and exhausted T cells (Tex)—underlies the reduced efficacy of immune checkpoint inhibitors (ICIs) observed in NSCLC patients with bone metastases [3, 4]. Indeed, a clinical trial has already linked bone metastases to poor clinical outcomes in ICI-treated NSCLC patients [5]. 

Within the TME, cancer-associated fibroblasts (CAFs) and tumor-associated macrophages (TAMs) are key orchestrators of tumor progression and immune suppression [6, 7]. High CAF abundance in solid tumors generally correlates with worse outcomes: tumors with a fibrotic, CAF-rich stroma tend to exclude cytotoxic immune cells and resist treatment. CAFs secrete extracellular matrix (ECM) proteins and immunomodulatory factors, creating physical and chemical barriers to T cell infiltration and fostering an immunosuppressive environment [8]. Notably, a TGF-β-driven CAF gene signature in stromal cells has been identified as a causal determinant of checkpoint immunotherapy failure in multiple cancers [9]. TAMs likewise promote metastasis and suppress immune responses via cytokine secretion and crosstalk with other cells [10]. For example, CAF–macrophage interactions can recruit monocytes and induce immunosuppressive TAM phenotypes, compounding immune evasion [11]. Consistent with this, heavy TAM infiltration often accompanies increased Treg levels in tumors, as TAM-derived chemokines (e.g., CCL22) actively recruit Tregs into the TME [12]. Despite their abundance, CAFs are highly heterogeneous in origin and function. Various precursors have been documented—including resident fibroblasts, pericytes, and even epithelial or endothelial cells undergoing mesenchymal transition [13, 14]—and emerging evidence suggests that some CAF subsets may derive from macrophages [15, 16]. How these distinct CAF subpopulations evolve during NSCLC progression, particularly in bone metastases, and what immunologic roles they play are still unclear.

Over the past decade, a process termed macrophage–myofibroblast transition (MMT) has gained recognition in both fibrosis and cancer [15, 17]. MMT describes the transdifferentiation of macrophages into myofibroblasts, wherein a macrophage acquires fibroblast markers like α-smooth muscle actin (α-SMA) while often downregulating its own macrophage markers. Previous studies in human renal fibrosis demonstrated that cells co-expressing macrophage markers (CD68, CD206) and myofibroblast markers (α-SMA) accumulate in active fibrotic lesions, suggesting that alternatively activated macrophages can directly convert into collagen-producing myofibroblasts in vivo [18]. In cancer, analogous transdifferentiation events are postulated to create pro-tumorigenic stroma. A recent single-cell analysis of NSCLC provided evidence of a macrophage-origin CAF subset (termed “MMT cells”) that was associated with increased CAF abundance and poorer patient survival. Fate-mapping and trajectory tracing confirmed that TAMs can transition into CAFs within tumors, and that disrupting TGF-β1/Smad3/Runx1 signaling in macrophages blocks this transition, thereby attenuating CAF accumulation and tumor progression [15, 19]. These findings establish MMT as a novel mechanism of CAF generation in cancer and point to its potential as a therapeutic target.

Despite these advances, the immunological impact of MMT-derived CAFs in cancer remains incompletely understood. Macrophage-lineage CAFs might wield unique immunoregulatory capabilities that shape the tumor immune landscape. Supporting this notion, certain CAF subsets have been shown to actively modulate T cells. For instance, in pancreatic ductal adenocarcinoma, a subset of CAFs with high MHC class II expression can present antigens to CD4⁺ T cells without co-stimulatory signals, thereby driving naïve CD4⁺ cells to differentiate into FOXP3⁺ Treg [20]. Similarly, in metastatic ovarian cancer, tumors enriched in Activin A–secreting CAFs exhibit high Treg infiltration, indicating that these fibroblasts help establish an immunosuppressive niche [21]. These observations suggest that analogous mechanisms may operate in metastatic lung cancer–namely, that unique CAF populations in NSCLC bone metastases promote Treg-mediated immunosuppression and T cell dysfunction. However, it remains unknown whether macrophage-derived CAFs contribute to immune escape in NSCLC metastases, and the key molecular regulators enabling macrophage-to-myofibroblast conversion in the metastatic setting have yet to be defined.

To address these gaps, we investigated CAF heterogeneity and macrophage plasticity in NSCLC bone metastases, focusing on immune remodeling within established lesions after metastatic colonization rather than seeding. By integrating single-cell transcriptomes from primary lung tumors and bone metastases, we resolved seven CAF subpopulations and identified a distinct subset co-expressing macrophage markers (e.g., CD68), MHC class II genes (e.g., CD74), and myofibroblast features (e.g., α-SMA), which was markedly enriched in bone lesions. Orthogonal analyses supported that this subset represents an MMT-linked, macrophage-lineage CAF phenotype (CAF_7); bulk deconvolution further associated higher CAF_7 abundance with poorer survival in LUAD/LUSC and similar unfavorable trends across additional cohorts. To model this transition from a standardized starting population, we used bone marrow–derived macrophages (BMDMs) and applied two complementary induction contexts: LLC-conditioned medium to recapitulate the phenotype in a tumor-mimicking secretome, and recombinant TGF-β1 as a defined stimulus for pathway-controlled perturbation; this dual-context design helps limit CM-driven macrophage programs unrelated to MMT [15, 19, 22–25]. We found that expansion of these MMT-derived CAFs in bone lesions is closely associated with an immunosuppressive microenvironment characterized by abundant regulatory T cells and exhausted CD8⁺ T cells. Through regulatory network analysis, we further identified the transcription factor ASCL2 as a central regulator of this transition. Functional experiments confirmed that inhibiting ASCL2 in macrophages blocks their conversion into CAFs; this intervention limits the formation of immunosuppressive stroma, restores effective anti-tumor T cell responses, and suppresses metastatic tumor growth. Mechanistically, to link ASCL2 to a downstream effector, we interrogated an ASCL2–Il6ra/IL6R regulatory axis using promoter-reporter assays and ChIP-based occupancy assays, and tested Il6ra function in macrophages in vivo. Notably, macrophage-specific ASCL2 inhibition also synergizes with PD-1 checkpoint blockade, leading to superior tumor control and prolonged survival in preclinical models. In summary, our study reveals that lung cancer bone metastases co-opt macrophages through ASCL2-driven MMT to generate immunosuppressive CAFs, and it highlights ASCL2 as a promising therapeutic target for reprogramming the metastatic microenvironment and enhancing immunotherapy efficacy.

## Methods

### Sample collection and patient characteristics

Bone metastasis samples were collected from 10 patients with pathologically confirmed NSCLC bone metastases for single-cell RNA sequencing analysis, following approval from the Ethics Committee of Guangdong Provincial People’s Hospital. Written informed consent was obtained from all participants. Among the 10 patients, six had not received any antitumor therapy prior to surgical resection. Patient ages ranged from 52 to 73 years, with a median age of 69 years. Detailed clinical and molecular information—including age, sex, smoking history, histological subtype, TNM stage, metastasis site, EGFR status, and PD-L1 expression—is summarized in Supplementary Table 1.

### Single-cell RNA sequencing and data processing

Lung cancer bone metastatic samples were obtained and processed within 2 h of surgical resection. Each specimen was mechanically dissociated and enzymatically digested using a tumor dissociation kit (Miltenyi Biotec, Germany) following the manufacturer’s protocol. Single-cell suspensions were filtered through a 40 μm cell strainer, and dead cells were removed using a Dead Cell Removal Kit (Miltenyi Biotec) to ensure high viability (> 85%) prior to library preparation.

Single-cell transcriptomic profiling was performed using the Chromium Single Cell 3′ Gene Expression platform (10× Genomics, Pleasanton, CA, USA). Briefly, approximately 8,000–12,000 viable cells per sample were loaded onto a Chromium Controller to generate single-cell gel beads-in-emulsion (GEMs) using the Chromium Next GEM Single Cell 3′ Reagent Kit v3.1 (10× Genomics), according to the manufacturer’s instructions. Reverse transcription and cDNA amplification were conducted within the droplets, followed by library construction. Final libraries were quality-checked using an Agilent Bioanalyzer and quantified via Qubit fluorometry.

Libraries were sequenced on the Illumina NovaSeq 6000 platform using a read configuration recommended for the 10× Genomics Chromium Single Cell 3′ Gene Expression assay, targeting a minimum depth of ≥ 50,000 reads per cell. The raw base call files were processed using the Cell Ranger software (v6.1.2, 10× Genomics), which performed read alignment to the human reference genome (GRCh38), transcript counting, and gene-cell barcode demultiplexing. The resulting gene expression matrices were used for downstream analyses.

### Initial quality control and dataset integration

To ensure data integrity and reduce technical noise, initial quality control was performed on both our in-house bone metastatic NSCLC single-cell RNA sequencing (scRNA-seq) dataset and two publicly available primary NSCLC scRNA-seq datasets (Wu et al., 2021; Zhang et al., 2022) [26, 27]. Filtering was conducted using the Seurat R package (v4.3.0). For each dataset, low-quality cells were excluded based on the following standardized criteria: (1) cells with fewer than 200 or more than 5,000 detected genes (nFeature_RNA) were discarded to eliminate empty droplets and likely doublets, respectively; (2) cells with mitochondrial transcript content exceeding 30% (percent.mt > 30) were removed to exclude low-quality or apoptotic cells; and (3) cells with excessive total UMI counts (> 30,000; nCount_RNA) were excluded to reduce multiplet contamination. Additionally, genes detected in fewer than 3 cells across the integrated dataset were removed to minimize background noise.

Following sample-specific filtering and normalization, all datasets were integrated using the Harmony algorithm for batch effect correction. Harmony was implemented after principal component analysis (PCA) to align shared biological variation across datasets while removing technical confounders. This approach effectively mitigated batch effects among the three datasets, enabling unified downstream analyses including clustering, developmental trajectory inference, and intercellular communication modeling.

### Doublet identification and removal

Putative doublets in the bone metastatic NSCLC scRNA-seq dataset were identified and removed using the DoubletFinder package in R. After standard Seurat preprocessing, including normalization, identification of highly variable genes, data scaling, and principal component analysis (PCA), artificial doublets were generated and integrated with the observed single-cell profiles to model doublet-enriched transcriptional states. Cells predicted as doublets were excluded from subsequent analyses.

### Visualization and cell type annotation

To visualize cellular heterogeneity, we applied t-distributed stochastic neighbor embedding (t-SNE) in Seurat to project high-dimensional transcriptomic data into two dimensions, enabling clear separation of transcriptionally distinct cell populations. Cell type annotation was performed using a combined approach. SingleR was first used to assign preliminary identities based on transcriptomic similarity to reference datasets of purified cell types [28]. These annotations were then refined by cross-validating with canonical marker genes, such as CLDN5, VWF, and PECAM1 for endothelial cells; COL1A1, LUM, and DCN for cancer-associated fibroblasts; CD3D, CD3E, and CD3G for T cells; CD79A and CD79B for B cells; CD68, CD163, and CD14 for macrophages; CSF3R, S100A8, and S100A9 for neutrophils; and GATA2, TPSAB1, and TPSB2 for mast cells. This integrative strategy enabled accurate classification of major cell types within the tumor and metastatic microenvironment, supporting robust downstream analyses.

### Cell–cell communication analysis

Cell–cell communication analysis was conducted using the CellChat R package [29], which enables systematic inference of intercellular signaling networks by leveraging a curated database of ligand–receptor interactions. The normalized single-cell transcriptomic matrix and Seurat-derived cell type annotations were used as input, with low-abundance populations excluded to ensure analytical stringency. Utilizing the CellChatDB.human reference database, probabilistic modeling was applied to estimate communication likelihood between cell types based on co-expression of ligands and their cognate receptors. Default parameters were employed for signaling inference, and key signaling sources and targets were delineated via the identifyCommunicationPatterns and computeNetAnalysis_signalingRole functions. To visualize the overall communication topology, interaction probabilities across cell types were mapped, enabling an intuitive representation of intercellular signaling landscapes.

### Pseudotime trajectory analysis

Pseudotime analysis was conducted using the Monocle 2 R package to infer dynamic cell state transitions [30]. Normalized gene expression data and cell identities processed via Seurat were used as input. Highly variable genes were selected for trajectory construction. Dimensionality reduction was performed using the DDRTree algorithm, and cells were ordered along pseudotime using the orderCells function. The trajectory root was defined based on known biological markers. Genes with significant changes along the pseudotime were identified and visualized to explore dynamic transcriptional programs.

To further delineate global lineage relationships and infer fate bias among cell subpopulations, we applied Partition-based Graph Abstraction (PAGA) using the Scanpy package. PAGA constructs a connectivity graph between clusters by computing the confidence of transitions based on transcriptomic similarity. This graph-based abstraction complements Monocle 2’s trajectory by providing a coarse-grained overview of cell–cell relationships and helps identify potential lineage bifurcations or convergence events, particularly within the CAF_7 or myeloid compartment.

### Regulon and transcription factor activity analysis

To investigate transcription factor (TF) activity and reconstruct gene regulatory networks at single-cell resolution, we utilized the pySCENIC pipeline on normalized single-cell RNA-sequencing data [31], preserving cell identity annotations from Seurat for downstream interpretation. The analysis comprised three core steps: (1) detection of co-expressed gene modules using GRNBoost2; (2) refinement of these modules into transcription factor regulons through motif enrichment analysis based on the cisTarget motif database; and (3) quantification of regulon activity in individual cells using the AUCell algorithm. This integrative approach enabled us to map cell type-specific regulatory programs and uncover candidate master regulators associated with distinct cellular phenotypes or dynamic transitions. All parameters were set to default unless otherwise specified.

### Estimation of CAF_7 abundance and prognostic analysis across cancer types

To estimate the relative abundance of the CAF_7 subpopulation across multiple cancer types, we applied CIBERSORTx (https://cibersortx.stanford.edu/) to bulk RNA-seq datasets retrieved from The Cancer Genome Atlas (TCGA) [32]. A single-cell–derived signature matrix was constructed based on the annotated CAF_7 gene expression profile from our single-cell dataset. Batch correction was enabled to account for technical variation across samples.

CAF_7 fractions were inferred for each tumor sample using the CIBERSORTx high-resolution mode, with 100 permutations and quantile normalization disabled for RNA-seq input. Samples with deconvolution p-values < 0.05 were retained for downstream analysis.

To evaluate the prognostic relevance of CAF_7 abundance, patients in each cancer cohort were stratified into CAF_7_high and CAF_7_low groups based on the inferred CAF_7 fraction. Kaplan–Meier survival analysis and log-rank tests were performed to assess differences in overall survival (OS) between groups. Statistical analyses were conducted using the R packages survival and survminer, and *p* < 0.05 was considered statistically significant.

### Pan-cancer transcriptomic and survival analysis

To explore the correlation and prognostic significance of gene expression signatures across multiple cancer types, we employed the GEPIA (http://gepia2.cancer-pku.cn/#correlation) and KMplot (https://kmplot.com) online platforms [33, 34], both based on TCGA pan-cancer transcriptomic and clinical data. Custom gene signatures were input into GEPIA to assess pairwise correlations between transcriptional programs. Specifically, the ASCL2–TF module was defined by ASCL2, FCGR2B, IL6R, and SASH3, and the immune signatures were defined as the Treg signature and the exhausted T cell signature, with the full gene lists provided in Supplementary Table 6. Correlation analyses were performed in GEPIA across all available TCGA cancer cohorts (*n* = 33) using the platform’s implementation for composite signature expression. For survival analysis, KMplot was used to evaluate the prognostic value of individual genes, with patients stratified into high- and low-expression groups based on the platform’s auto-selected best cutoff. Kaplan–Meier curves, hazard ratios (HRs), and log-rank p-values were generated, and *p* < 0.05 was considered statistically significant.

### Cell culture, treatment, and T cell co-culture assay

#### Cell culture and BMDM differentiation

Lewis lung carcinoma (LLC) cells were maintained in DMEM/F12 medium supplemented with 10% fetal bovine serum (FBS), 100 U/mL penicillin, and 100 µg/mL streptomycin at 37 °C in a humidified incubator containing 5% CO₂. BMDMs were isolated from the femurs, tibias, and iliac bones of C57BL/6 mice and differentiated for 7 days in DMEM containing 10% heat-inactivated FBS, antibiotics, and 50 ng/mL murine macrophage colony-stimulating factor (M-CSF, Gibco), following established protocols [19]. BMDMs were selected as a standardized primary macrophage starting population to enable controlled induction and quantitative assessment of macrophage-to-myofibroblast transition (MMT), thereby providing a reproducible methodological link between the macrophage origin and the CAF_7 endpoint described in this study. The LLC-CM was obtained by incubating LLC cells overnight in serum‐free DMEM/F12, followed by filtration using a 0.2 μm nylon membrane [19]. 

#### Quantification of SPP1 secretion by LLC-CM– or TGF-β1–programmed BMDMs (ELISA)

To determine whether programmed/transitioned BMDMs acquire a CAF_7-like secretome, we quantified secreted SPP1 in BMDM-conditioned media by sandwich ELISA. To ensure that the measured proteins reflect newly synthesized and released factors from CAF_7-like cells, rather than residual cytokines carried over from the inducing stimuli, we applied the same standardized two-phase conditioning strategy to both induction contexts. In the defined MMT setting, this protocol is identical except that LLC-CM is replaced by recombinant TGF-β1 (5 ng/mL).

Programming/induction phase: Differentiated BMDMs were subjected to one of the following induction conditions for 5 consecutive days: (i) freshly prepared LLC-conditioned medium (LLC-CM), or (ii) recombinant TGF-β1 (5 ng/mL) to induce MMT. Where indicated, BMDMs in the TGF-β1–MMT condition were transfected with siRNA (negative control, siNC; or ASCL2-targeting siRNA, siASCL2) to generate siNC-BMDMs or siASCL2-BMDMs, respectively. Control BMDMs were maintained in matched fresh medium under otherwise identical conditions.

Washout and secretion phase: After the 5-day programming/induction period, cells were washed extensively with pre-warmed PBS (≥ 3 times) and then incubated in fresh complete medium for an additional 12–24 h to allow accumulation of newly secreted proteins. Supernatants collected during this secretion phase were designated as “BMDM-conditioned media” and used for ELISA.

Sample clarification and ELISA: Conditioned media were clarified by sequential centrifugation (500 × g, 5 min; then 10 000 × g, 10 min, 4 °C) and 0.22-µm filtration. SPP1 concentrations were measured using commercially available sandwich ELISA kits (R&D Systems, Bio-Techne) according to the manufacturers’ instructions. Standards and samples were loaded in technical duplicates (50–100 µL/well, as recommended by the kits), developed, and read at 450 nm with 570 nm reference on a microplate reader. Concentrations were calculated from standard curves and expressed as pg/mL.

Only secretion-phase supernatants collected after extensive washout and fresh-medium incubation were used for quantification, thereby minimizing stimulus carryover and ensuring that measured SPP1 predominantly represents CAF_7-like cell–derived de novo secretion.

#### Experimental groups and TGF-β1–induced MMT

To assess whether ASCL2 knockdown alters macrophage-to-myofibroblast transition (MMT) and modulates T cell responses, BMDMs were divided into three groups: (1) untreated controls (no TGF-β1), (2) siNC-BMDMs (transfected with non-targeting siRNA + TGF-β1), and (3) siASCL2-BMDMs (transfected with ASCL2-targeting siRNA + TGF-β1). Transfections were performed using Lipofectamine™ RNAiMAX (Thermo Fisher Scientific), and TGF-β1 (5 ng/mL) was applied for 5 consecutive days to induce MMT [15, 19, 22]. Each group of cells was subsequently processed for downstream assays. The siRNA sequences used for ASCL2 knockdown and negative control are listed in Supplementary Table 2.

#### ASCL2^res rescue in TGF-β1–induced MMT in BMDMs

To validate the specificity of the siASCL2 phenotype and to determine whether restoring ASCL2 is sufficient to reinstate the MMT-associated molecular program under inductive conditions, an RNAi-resistant ASCL2 re-expression construct (ASCL2^res; “A2-res”) was used in BMDMs subjected to TGF-β1–induced MMT. The ASCL2^res construct was engineered to be resistant to the siASCL2 sequence by introducing silent substitutions within the siRNA target region, thereby preserving the ASCL2 amino-acid sequence while preventing siRNA-mediated degradation.

Differentiated BMDMs were co-transfected with siASCL2 together with either an empty vector (EV) or ASCL2^res (A2-res). In parallel, siNC + EV served as the control condition. Following transfection, cells were stimulated with recombinant TGF-β1 (5 ng/mL) for 5 consecutive days to induce MMT, with matched medium refreshment according to the standard induction protocol used in this study. At the end of induction, cells were harvested for protein analysis.

For readouts, whole-cell lysates were prepared and subjected to Western blotting to confirm ASCL2^res re-expression and to assess associated marker changes, including IL6R, macrophage-lineage/MHC-II–associated markers (F4/80, CD68, CD74), MMT/CAF markers (FAP, α-SMA, VEGF), and immunoregulatory proteins (MRC1, HAVCR2).

#### T cell isolation

CD4⁺ and CD8⁺ T cells were isolated from the spleens of C57BL/6 mice by column-free immunomagnetic negative selection using the EasySep™ Mouse CD4⁺/CD8⁺ T Cell Isolation Kits (STEMCELL Technologies), following the manufacturer’s instructions. This approach yields untouched T cells suitable for downstream flow cytometry, culture, and functional assays, with minimal perturbation of surface phenotype and transcriptomic profiles. Depletion panels: the CD4 kit removes CD11b, CD45R, Ter119, CD8a, CD49b, CD19, CD11c, TCRγδ, CD24; the CD8 kit removes CD11b, CD45R, Ter119, CD4, CD49b, CD19, CD11c, TCRγδ, CD24.

#### T cell co-culture assay

BMDMs in the TGF-β1–MMT condition were transfected with siRNA (siNC; siASCL2) to generate siNC-BMDMs or siASCL2-BMDMs, respectively. Control BMDMs were maintained in matched fresh medium under otherwise identical conditions. Before co-culture, BMDMs were washed three times with PBS. For the co-culture assay, 2 × 10⁴ BMDMs were seeded with 5 × 10⁴ freshly isolated CD4⁺ or CD8⁺ T cells in complete DMEM containing 10% FBS in 96-well round-bottom plates. Co-cultures were incubated for 18 h. T cells were then collected, washed three times with PBS, and subjected to downstream phenotypic and functional analyses.

### In vivo functional assays and therapeutic evaluation in a murine lung cancer bone metastasis model

#### Murine intratibial lung cancer bone metastasis model

To elucidate the functional role of ASCL2 in lung cancer bone metastasis and its regulation of macrophage-to-myofibroblast transition (MMT), an immunocompetent murine model of lung cancer bone metastasis was established as previously described [35–37]. Briefly, C57BL/6 mice were anesthetized with 1.25% tribromoethanol via intraperitoneal injection, and a small incision was made to expose the patellar ligament. A 25-gauge needle was first used to create an entry route through the patellar ligament into the tibial marrow cavity. The needle was then replaced with a 10 µL microinjection syringe containing a suspension of LLC cells, and 5 × 10⁵ Lewis lung carcinoma (LLC) cells were slowly injected into the tibial marrow cavity to simulate metastatic colonization of the bone. After injection, the incision was covered with a gelatin sponge to prevent leakage [36]. For sham-operated controls, mice underwent identical anesthesia, surgical exposure, and tibial cortical entry, followed by intramedullary injection of an equal volume of sterile PBS instead of tumor cells. Tumor engraftment was confirmed by in vivo bioluminescence imaging prior to downstream analyses. Importantly, our in-vivo analyses were confined to post–colonization immune–stromal remodeling within established bone lesions, rather than to dissemination or initial seeding. All animal experiments were approved by the Ethics Committee of Guangdong Provincial People’s Hospital. For endogenous lineage-tracing experiments, LysM-Cre; Rosa26-tdTomato mice were used (parental strains: Jackson Laboratory Stock Nos. 004781 and 007914).

#### Comparative analysis of bone versus subcutaneous tumors

To determine whether the bone metastatic niche is associated with increased ASCL2 activity, paired tumor models were established in C57BL/6 mice. For the bone metastasis (BM) model, 5 × 10⁵ Lewis lung carcinoma (LLC) cells were injected into the tibial marrow cavity as described above. For the subcutaneous (SC) model, the same number of LLC cells was injected into the hindlimb subcutis of syngeneic mice under 1.25% tribromoethanol anesthesia. After tumor establishment was confirmed by bioluminescence imaging at day 14–17 post-implantation, tissues were harvested for downstream analyses.

#### Local siRNA administration

To assess the role of ASCL2 in vivo, mice were randomly divided into groups receiving peritumoral injections of siNC or siASCL2, each complexed with Invivofectamine™ 3.0 (Thermo Fisher Scientific) according to the manufacturer’s instructions. After tumor establishment was confirmed by in vivo bioluminescence imaging (IVIS Spectrum, PerkinElmer), typically 5–7 days after intratibial implantation, 40 µL of siRNA complexes (1 µg siRNA per mouse) were administered locally to the peritumoral region. Prior to tissue harvest, in vivo bioluminescence imaging was conducted using the IVIS Spectrum system to assess tumor burden.

#### Adoptive transfer of ASCL2-silenced macrophages

To further investigate the cell-intrinsic role of ASCL2 in MMT in vivo, BMDMs transfected with either siNC or siASCL2 were used for adoptive transfer. Recipient C57BL/6 mice were pretreated with clodronate liposomes (200 µL per mouse, i.v.; YEASEN) to deplete endogenous macrophages 2 days before tumor inoculation. On the day of LLC implantation, LLC cells were co-injected with siNC-BMDMs or siASCL2-BMDMs (1:1 ratio; total 5 × 10⁵ cells per mouse), with a PBS co-injection control using the same LLC input as the BMDM co-injection groups, into the tibia under anesthesia with 1.25% tribromoethanol via intraperitoneal injection [15, 19, 38]. Prior to tissue harvest, in vivo bioluminescence imaging was conducted using the IVIS Spectrum system to assess tumor burden. The same macrophage depletion–reconstitution framework was additionally applied for downstream validation using Il6ra-silenced BMDMs. For donor-tracing experiments, BMDMs were first labeled by GFP transfection and then introduced under the same macrophage depletion–reconstitution framework, with co-injection into the tibia together with LLC cells at the same ratio.

#### Evaluation of ASCL2-PD-1 combination therapy

To evaluate the therapeutic synergy between ASCL2 knockdown and PD-1 immune checkpoint blockade, tumor-bearing mice were randomly assigned into four treatment groups after tumor establishment: (1) siNC with IgG2a isotype control (siNC + IgG), (2) siNC with anti-PD-1 antibody (siNC + αPD-1), (3) siASCL2 with IgG2a isotype control (siASCL2 + IgG), and (4) siASCL2 with anti-PD-1 antibody (siASCL2 + αPD-1). Peritumoral siRNA administration using Invivofectamine™ 3.0 was performed every 3 days for four doses. Anti-PD-1 monoclonal antibody or IgG2a isotype control (RMP1-14, Bio X Cell) was administered intraperitoneally at 200 µg per mouse every 4 days for four doses [39]. Tumor burden was quantified by in vivo bioluminescence imaging after completion of the final dose to evaluate treatment response, and survival was monitored until humane endpoints were reached or until the predefined study endpoint, whichever occurred first.

### Quantitative Real-Time PCR (qPCR)

Total RNA was extracted from in vivo tumor tissues and in vitro cultured cells using TRIzol™ reagent (Invitrogen, Thermo Fisher Scientific) according to the manufacturer’s protocol. RNA concentration and purity were assessed using a NanoDrop 2000 spectrophotometer (Thermo Fisher Scientific). For each sample, 1 µg of total RNA was reverse-transcribed into cDNA using the PrimeScript™ RT Master Mix (Takara).

Quantitative real-time PCR was performed using TB Green^®^ Premix Ex Taq™ II (Takara) on a LightCycler^®^ 480 system (Roche). The 2^–ΔΔCt method was used to determine relative gene expression levels, normalized to GAPDH as the internal control. All reactions were carried out in triplicate, and melt curve analyses were performed to confirm the specificity of amplification. Primer sequences used in this study are listed in Supplementary Table 3.

### Operational definition and terminology of CAF_7 and CAF_7-like cells

In this study, CAF_7 refers to the transcriptionally defined CAF subcluster identified by scRNA-seq and characterized by the co-enrichment of myofibroblastic/fibroblastic, macrophage-lineage, and MHC class II-associated programs. Because no single two-marker combination can fully capture this transcriptional state across all downstream validation settings, the term CAF_7-like cells is used to describe marker-defined populations in experimental validation.

For fixed-tissue and endpoint validation assays, CAF_7-like cells were primarily defined as α-SMA⁺CD68⁺ double-positive cells, reflecting the coexistence of myofibroblastic and macrophage-lineage features. α-SMA⁺CD74⁺ was used as a complementary marker combination to reflect myofibroblastic and MHC class II-associated features. Thus, throughout the revised manuscript, CAF_7 denotes the scRNA-seq-defined transcriptional cluster, whereas CAF_7-like cells denote marker-defined populations used for downstream validation.

### Flow cytometry analysis of in vitro and in vivo samples

Flow cytometry was conducted on both in vitro cultured cells and single-cell suspensions derived from tumor-bearing tibiae of lung cancer bone metastasis models. Tumor tissues were enzymatically and mechanically dissociated, then filtered through a 40 μm nylon mesh to obtain single-cell suspensions. Cultured cells were collected and washed with PBS. All samples were stained with DAPI to exclude non-viable cells. CAF_7-like cells were identified based on co-expression of α-SMA and CD68. Regulatory T cells (Tregs) were defined as CD45⁺CD3⁺CD4⁺CD25⁺ cells with intracellular FOXP3 positivity. Exhausted CD8⁺ T cells were characterized by co-expression of CD45, CD3, CD8, PD-1, and TIM-3. All antibodies were used at optimized concentrations and incubated for 30 min at 4 °C in the dark. Flow cytometry acquisition was performed on a BD flow cytometer (BD Biosciences), and data were analyzed using FlowJo software version 10 (Tree Star). Detailed information on fluorochrome-conjugated antibodies is listed in Supplementary Table 5.

### Flow cytometric sorting of donor-derived GFP⁺FAP⁺/GFP⁺FAP⁻ cells and tdTomato⁺FAP⁺/tdTomato⁺FAP⁻ cells

Because intracellular α-SMA staining is incompatible with the recovery of viable cells for downstream assays, surface FAP was used here as a surrogate marker to enrich CAF_7-like fractions. Bone metastatic lesions were dissociated into single-cell suspensions and passed through a 40 μm cell strainer. Immediately before sorting, cells were stained with an anti-FAP antibody and DAPI. Flow-cytometric sorting was performed by sequential gating to exclude debris, select singlets, and retain viable DAPI-negative cells. For donor-tracing experiments, GFP⁺ cells were then identified and further separated into GFP⁺FAP⁺ and GFP⁺FAP⁻ fractions. For endogenous lineage-tracing experiments using LysM-Cre; Rosa26-tdTomato mice, tdTomato⁺ cells were similarly identified after the same gating steps and further separated into tdTomato⁺FAP⁺ and tdTomato⁺FAP⁻ fractions. Sorted cells were collected directly into complete culture medium for downstream experiments.

### Immunofluorescence staining of cultured cells, murine tumors, and human primary and bone metastatic lung cancer tissues

Immunofluorescence staining was performed on (1) in vitro–cultured macrophages, (2) tumor tissues obtained from murine models of lung cancer bone metastasis, and (3) human primary lung cancer specimens and lung cancer bone metastasis specimens to evaluate the expression and spatial distribution of key immune and stromal markers.

Cultured BMDMs were fixed with 4% paraformaldehyde for 15 min, permeabilized with 0.3% Triton X-100, and blocked with 5% bovine serum albumin (BSA). Cells were then incubated overnight at 4 °C with primary antibodies against F4/80 and α-SMA, followed by fluorophore-conjugated secondary antibodies and nuclear counterstaining using DAPI.

For murine and human tissues, samples were fixed in 4% paraformaldehyde, embedded in O.C.T. compound, cryosectioned at 8 μm thickness, and processed with the same permeabilization and blocking steps. Sections were incubated overnight at 4 °C with primary antibodies targeting CD68, α-SMA, CD74, CD4, FOXP3, CD8, PD-1, HAVCR2 (TIM-3), TCF-1, and ASCL2. After secondary antibody incubation and DAPI staining, slides were mounted using antifade mounting medium.

Fluorescent images were acquired using a Leica SP8 confocal microscope (Leica Microsystems) and analyzed with ImageJ software. A comprehensive list of primary and secondary antibodies used in these experiments is provided in Supplementary Table 5.

### Western blot

Protein lysates were prepared from both in vitro–cultured cells and tumor tissues derived from the lung cancer bone metastasis model using RIPA lysis buffer supplemented with protease and phosphatase inhibitor cocktails. Protein concentrations were quantified using a BCA Protein Assay Kit (Thermo Fisher Scientific) in accordance with the manufacturer’s protocol. Equal amounts of total protein were separated via SDS–polyacrylamide gel electrophoresis (SDS-PAGE) and transferred onto polyvinylidene fluoride (PVDF) membranes (Millipore).

Membranes were blocked with 5% non-fat dry milk in TBST (Tris-buffered saline with 0.1% Tween-20) for 1 h at room temperature, followed by overnight incubation at 4 °C with primary antibodies against the following targets: F4/80, FAP, α-SMA, VEGF, IL6R, CD68, CD74, MRC1, HAVCR2, ASCL2, and GAPDH. After washing with TBST, membranes were incubated with species-appropriate HRP-conjugated secondary antibodies for 1 h at room temperature.

Immunoreactive protein bands were visualized using an enhanced chemiluminescence (ECL) detection kit (Thermo Fisher Scientific) and imaged with a Bio-Rad chemiluminescence detection system. A complete list of all primary and secondary antibodies used is provided in Supplementary Table 5.

### Dual-luciferase reporter analysis

Using pcDNA3.1-based plasmids, we assessed ASCL2-dependent transactivation of the Il6ra promoter. An ASCL2 expression construct (pcDNA3.1-ASCL2, wild-type) and its matched empty vector (EV) control were used for gain-of-function. The reporter plasmid was pGL3-basic carrying a mouse Il6ra promoter fragment upstream of the transcription start site (Il6ra-WT); empty pGL3-basic served as the vector control. Renilla luciferase (pRL) was co-transfected as an internal control for normalization.

HEK293T cells were plated in 24-well plates and co-transfected with the firefly reporter (pGL3-Il6ra-WT or pGL3-basic), pcDNA3.1-ASCL2 (or EV), and pRL using a lipid-based transfection reagent according to the manufacturer’s instructions. After 48 h, cells were lysed and assayed with the Promega Dual-Luciferase^®^ Reporter System on a luminometer. Results are based on three independent experiments.

### ChIP–qPCR analysis

To examine ASCL2 occupancy at the Il6ra promoter, chromatin immunoprecipitation coupled with qPCR (ChIP–qPCR) was performed in BMDMs. Briefly, BMDMs were cross-linked and lysed, and chromatin was fragmented by sonication. 10% of the sheared chromatin was reserved as the input control. Immunoprecipitation was carried out using an anti-ASCL2 antibody (1:50), with normal IgG included as a negative control. The immunoprecipitated DNA was purified using a Universal DNA Purification Kit (Tiangen, Beijing, China) and quantified by qPCR.

For the TGF-β1 or PBS pretreatment groups, BMDMs were treated with TGF-β1 or PBS for 48 h prior to ChIP. Putative ASCL2-binding motifs in the Il6ra locus were predicted using JASPAR, and a candidate regulatory region was selected for ChIP–qPCR. Primer sequences are provided in Supplementary Table 4.

### Collagen gel contraction assay

Freshly isolated GFP⁺FAP⁺/GFP⁺FAP⁻ or tdTomato⁺FAP⁺/tdTomato⁺FAP⁻ cells from dissociated bone metastatic lesions, as well as BMDMs under the indicated treatment conditions, were subjected to a collagen gel contraction assay. Cells were suspended on ice in neutralized type I collagen (Corning) at 2 mg/mL to generate cell-populated gels. The collagen-cell mixture was dispensed into culture plates and allowed to polymerize at 37 °C for 30 min. After polymerization, gels were gently detached from the well edges to permit free-floating contraction, overlaid with complete culture medium, and incubated at 37 °C in 5% CO₂. Gel images were acquired at 48 h, and gel contraction was quantified by measuring gel area with ImageJ. A greater reduction in gel area was interpreted as increased contractile capacity.

### CAF_7 module definition and signature scoring/cross-sample correlation analyses

#### CAF subset identification and CAF_7 differential-expression analysis

Single-cell RNA-seq data were processed and annotated as described above. CAFs were identified based on canonical fibroblast/stromal markers and subsequently reclustered to resolve CAF subpopulations (CAF_1–CAF_7). To stringently define CAF_7-enriched features, differential-expression analysis was performed comparing CAF_7 versus the pooled CAF_1–CAF_6 populations. Genes were considered CAF_7-enriched if they met all of the following criteria: avg_log2FC > 1, pct.1 > 0.3, and adjusted *P* < 0.01 (multiple testing corrected). This yielded a high-confidence CAF_7-enriched DEG pool (> 400 genes). For transparency, the complete CAF_7-enriched DEG list (DEG_CAF7_vs_otherCAFs) is provided in the Supplementary Materials.

#### Module-anchored CAF_7 marker framework and DotPlot visualization

Because the CAF_7-enriched DEG pool is broad and contains functionally overlapping genes, we adopted an identity-centered, module-based summarization strategy to improve interpretability. CAF_7-enriched genes were organized into four functional modules capturing the core phenotype dimensions of CAF_7: (i) macrophage-lineage/antigen-presentation (MHC-II) trace, (ii) secretome/chemokines, (iii) immune checkpoint/Treg-support, and (iv) ECM/myofibroblast. The module structure and the corresponding genes are shown in Fig. 2A. Module expression patterns were visualized using a DotPlot/summary panel across TAMs (“Macro”) and CAF_1–CAF_7. Dot size represents the fraction of expressing cells and dot color represents average expression.

#### CAF_7 signature scoring, sample-level aggregation, and correlation analyses

To test CAF_7-associated immune relationships at the sample level, we computed module scores using Seurat AddModuleScore with explicitly defined gene sets. For the CAF_7 score used in Fig. 3, we employed a CAF_7-specific immune-regulatory signature composed of the macrophage-lineage/MHC-II, secretome/chemokines, and checkpoint/Treg-support modules, while intentionally excluding broadly expressed CAF/ECM/myofibroblast genes (e.g., COL1A1/COL1A2, FAP, α-SMA) to maximize discriminability of the CAF_7-enriched program within the CAF compartment. The exact gene lists for CAF_7, Treg, and exhausted CD8 T-cell scoring are provided in Table S6.

Module scores were first computed at the single-cell level and then aggregated at the sample level (one CAF_7 score derived from CAFs, one Treg score derived from CD4⁺ T cells, and one exhaustion score derived from CD8⁺ T cells per sample) prior to correlation analysis, reducing bias from unequal cell numbers across samples. Correlations were evaluated at the between-sample level using appropriate correlation statistics as described in the Statistical Analysis section.

### Statistical analysis

Data are presented as the mean ± standard error of the mean (SEM). Statistical comparisons between two groups were performed using either a two-tailed unpaired or paired Student’s t-test, as appropriate. Comparisons among multiple groups were conducted using one-way analysis of variance (ANOVA) followed by Tukey’s post hoc test for normally distributed data, or non-parametric tests when assumptions of normality or homogeneity of variance were not met. Survival curves were compared using the log-rank (Mantel–Cox) test. Correlation analyses were performed using Spearman’s rank correlation coefficient. A p-value < 0.05 was considered statistically significant. All statistical analyses were performed using GraphPad Prism software (version 8.0) and R software.

## Results

### Identification and enrichment of CAF_7 across NSCLC progression and bone metastases

**Fig. 1:**
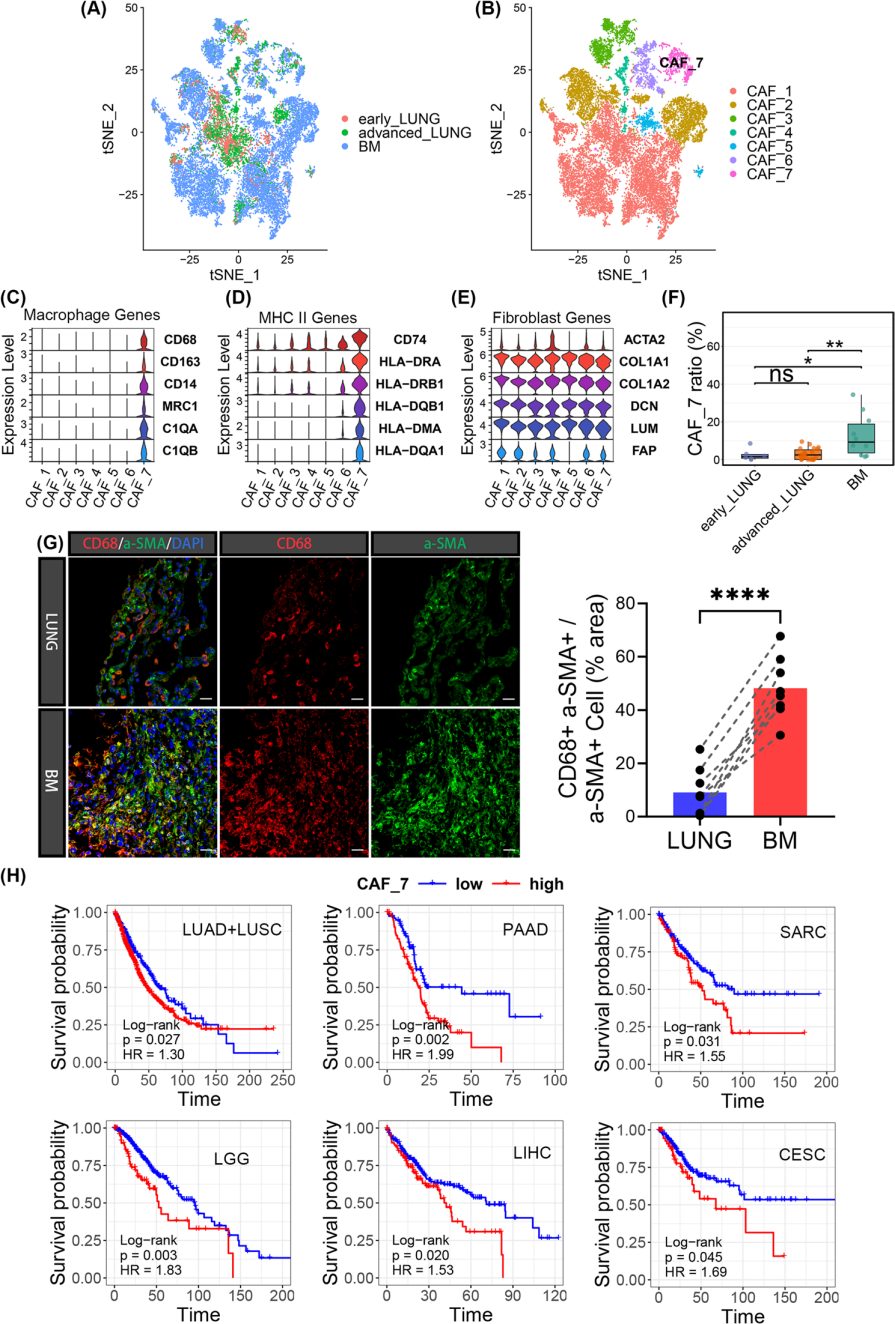
Identification and enrichment of CAF_7 across NSCLC progression and bone metastases. **A**, **B **t-SNE plots of integrated single-cell RNA-seq data from 10 bone metastases (BM), 22 early-stage primary tumors (early_LUNG), and 39 advanced-stage tumors (advanced_LUNG), colored by clinical group (**A**) and cancer-associated fibroblast (CAF) clusters (**B**). Seven CAF subsets (CAF_1–CAF_7) were identified. **C**–**E **Violin plots show gene expression across CAF subsets. CAF_7 co-expresses macrophage markers (CD68, CD163), MHC class II genes (e.g., CD74, HLA-DRA), and fibroblast markers (e.g., α-SMA, COL1A1, COL1A2), supporting its hybrid transcriptional profile. **F **Quantification of CAF_7 proportions among total CAFs shows a progressive increase from early_LUNG (n = 6) to advanced_LUNG (*n* = 22), with the highest enrichment observed in BM (*n* = 10). Only samples with more than 20 CAF cells were included to ensure statistical robustness. (*p < 0.05, **p < 0.01; ns, not significant). **G **Representative immunofluorescence staining of CD68 and α-SMA in paired LUNG and BM tissues (*n* = 8). CD68⁺α-SMA⁺ double-positive cells were used here as the primary marker combination for identifying CAF_7-like cells, capturing both macrophage-lineage and myofibroblastic features. CAF_7-like cells are enriched in BM regions. Quantification shows a significantly higher proportion of CD68⁺α-SMA⁺ CAF_7-like cells among total α-SMA⁺ CAFs in BM versus LUNG (paired t-test; ****p < 0.0001). Scale bar, 20 μm. Colors: CD68, red; α-SMA, green; DAPI, blue. **H **Kaplan–Meier survival analysis across six TCGA cancer cohorts using a bone metastasis–derived CAF_7 signature estimated by CIBERSORTx. Cox hazard ratios (HR; high vs low) are shown in each panel together with log-rank p values. Higher CAF_7 levels were associated with worse overall survival in lung adenocarcinoma and lung squamous cell carcinoma (LUAD/LUSC) (HR = 1.30; log-rank *p* = 0.027) and similarly unfavorable survival trends in pancreatic adenocarcinoma (PAAD), sarcoma (SARC), lower-grade glioma (LGG), liver hepatocellular carcinoma (LIHC), and cervical squamous cell carcinoma and endocervical adenocarcinoma (CESC).

To characterize the dynamic evolution of cancer-associated fibroblast (CAF) subsets during non-small cell lung cancer (NSCLC) progression, we integrated single-cell RNA sequencing data from 10 bone metastases and 61 primary NSCLC tumors (22 early-stage and 39 advanced-stage [all IIIB–IV, AJCC 8th edition]), revealing seven distinct CAF subpopulations (CAF_1–CAF_7) through unified clustering and t-SNE analysis (Fig. 1A, B). CAF_7 uniquely exhibited co-expression of macrophage markers (CD68, CD163), MHC class II genes (CD74, HLA-DRA), and classical fibroblast markers (α-SMA/ACTA2, COL1A1, COL1A2) (Fig. 1C–E). Notably, the abundance of CAF_7 gradually increased from early-stage tumors to advanced-stage tumors and was most pronounced in bone metastases (Fig. 1F). This trend was based on a filtered dataset that included only samples containing more than 20 CAF cells to ensure statistical rigor. Immunofluorescence analysis of paired primary lung and bone metastatic tissues from eight patients validated the marked enrichment of CAF_7-like cells (CD68⁺α-SMA⁺) within metastatic bone lesions, where the proportion of CAF_7-like cells relative to total α-SMA⁺ CAFs was significantly higher compared to primary tumors, suggesting an adaptive specialization of CAF_7-like cells in the bone microenvironment (Fig. 1G). Furthermore, Venn analysis demonstrated limited overlap (360 shared genes; Jaccard overlap = 22%) between the top 1000 highly expressed genes of CAF_7 from bone metastases and those from advanced primary tumors (Figure S1A, Supporting Information). Functional enrichment analysis revealed highly divergent biological processes (Jaccard overlap = 6.09%) between these CAF_7 populations, indicative of metastasis-specific transcriptional reprogramming (Figure S1B, Supporting Information). Finally, using a CAF_7 signature derived from bone metastases, CIBERSORTx deconvolution across bulk transcriptomic data from six TCGA cancer cohorts (LUAD/LUSC analyzed jointly, PAAD, SARC, LGG, LIHC, and CESC) showed that higher CAF_7 abundance was associated with worse overall survival in LUAD/LUSC (Cox HR [high vs. low] = 1.30; log-rank *p* = 0.027) and similarly unfavorable survival trends in the other cohorts (Fig. 1H). In contrast, when applying a CAF_7 signature derived from advanced-stage primary NSCLC tumors, the prognostic association was less consistent and limited to a subset of cancer types (Figure S1C, Supporting Information). These results suggest that CAF_7 populations arising in the bone metastatic context acquire distinct pro-tumor transcriptional features that may be associated with broader prognostic relevance across multiple cancer types than their counterparts in primary tumors.

### Pseudotime and experimental evidence support MMT-derived CAF_7 formation from TAMs

**Fig. 2:**
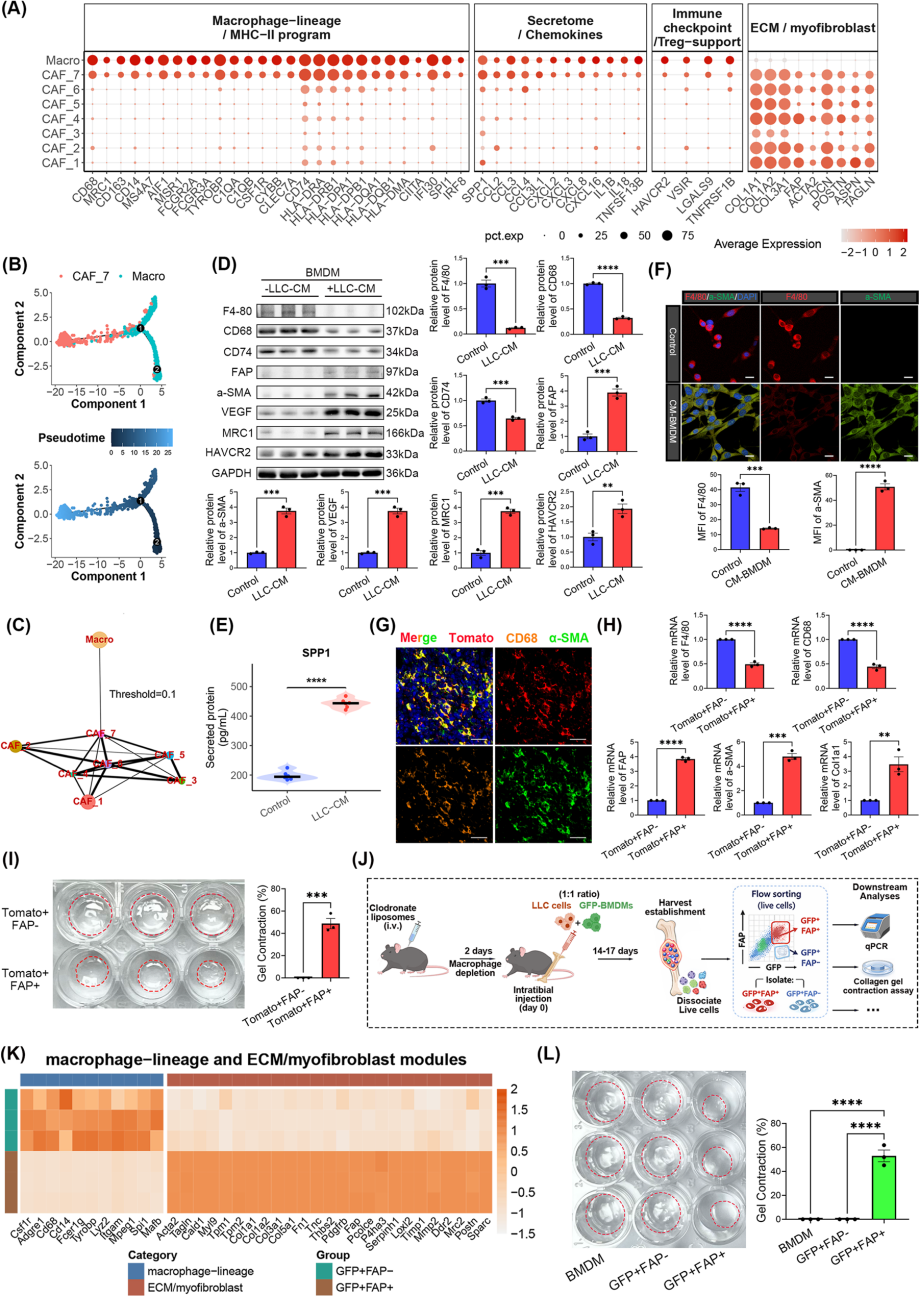
Pseudotime and experimental evidence support MMT-derived CAF_7 formation from TAMs. **A** DotPlot of CAF_7 marker modules derived from differential-expression analyses of CAF_7 versus CAF_1–CAF_6. Genes are grouped into four functional modules (macrophage-lineage/MHC-II program, secretome/chemokines, immune checkpoint/Treg-support, and ECM/myofibroblast) and displayed across TAMs (“Macro”) and CAF_1–CAF_7 subsets. Dot size denotes the fraction of expressing cells, and color denotes average expression. **B **Monocle 2 pseudotime analysis identifies a differentiation trajectory from TAMs (“Macro”) toward CAF_7, shown by cluster annotation (top) and pseudotime coloring (bottom). **C **PAGA analysis shows strong graph connectivity between TAMs (“Macro”) and CAF_7 (threshold = 0.1), supporting a continuous lineage relationship. **D **Western blot and densitometric quantification of BMDMs cultured with or without LLC-CM. LLC-CM decreases macrophage-associated proteins (F4/80, CD68, and CD74) and increases myofibroblast/CAF-associated markers (FAP, α-SMA, and VEGF) as well as immune regulation–related proteins (MRC1 and HAVCR2) (*n* = 3; **p < 0.01, ***p < 0.001, ****p < 0.0001). **E **ELISA measurement of secreted SPP1 in culture supernatants from control and LLC-CM–treated BMDMs (*n* = 6; ****p < 0.0001). **F **Immunofluorescence staining and quantitative analysis of bone marrow–derived macrophages (BMDMs) treated with or without Lewis lung carcinoma–conditioned medium (LLC-CM). LLC-CM exposure led to a marked decrease in F4/80 expression and a significant increase in α-SMA levels, accompanied by morphological changes consistent with a fibroblast-like phenotype (*n* = 3, ***p < 0.001, ****p < 0.0001; mean fluorescence intensity [MFI]). Scale bars, 20 μm. Colors: F4/80 red; α-SMA green; DAPI blue. **G **Immunofluorescence analysis of bone metastatic lesions from LysM-Cre;Rosa26-tdTomato mice. Representative images show tdTomato-labeled cells (red), CD68 (orange), α-SMA (green), and DAPI (blue). Merged images show tdTomato-labeled cells positive for both CD68 and α-SMA. Scale bars, 20 μm. **H **qPCR analysis of sorted tdTomato⁺FAP⁺ and tdTomato⁺FAP⁻ populations isolated from bone lesions. Compared with the tdTomato⁺FAP⁻ fraction, tdTomato⁺FAP⁺ cells showed increased expression of FAP, α-SMA, and Col1a1 together with reduced expression of F4/80 and CD68 (*n* = 3; **p < 0.01, ***p < 0.001, ****p < 0.0001). **I **Collagen gel contraction assay of sorted tdTomato⁺FAP⁻ and tdTomato⁺FAP⁺ cells. tdTomato⁺FAP⁺ cells exhibited greater gel contraction capacity than tdTomato⁺FAP⁻ cells (*n* = 3; ***p < 0.001). **J **Schematic of an in vivo donor-tracing experiment performed in a macrophage depletion/reconstitution intratibial bone metastasis model. Endogenous macrophages were depleted with clodronate liposomes, and GFP-labeled bone marrow–derived macrophages (BMDMs) were co-injected with LLC cells into the tibia at a 1:1 ratio. To permit recovery of viable cells for downstream analyses, surface FAP was used as a surrogate marker to sort GFP⁺FAP⁺ and GFP⁺FAP⁻ donor-derived populations from dissociated bone metastatic lesions. **K** Transcriptomic comparison of GFP⁺FAP⁺ and GFP⁺FAP⁻ populations. Compared with the GFP⁺FAP⁻ fraction, GFP⁺FAP⁺ cells showed attenuation of macrophage-lineage features and coordinated enrichment of a broader ECM/myofibroblast program. **L **Collagen gel contraction assay of BMDMs, GFP⁺FAP⁻ cells, and GFP⁺FAP⁺ cells. GFP⁺FAP⁺ cells exhibited greater gel contraction capacity than BMDMs and GFP⁺FAP⁻ cells, indicating acquisition of CAF-like contractile properties (*n* = 3; ****p < 0.0001)

To define CAF_7-specific features stringently and evaluate whether CAF_7 may arise from tumor-associated macrophages (TAMs) via macrophage-to-myofibroblast transition (MMT), we derived a CAF_7 marker panel from differential-expression analyses of CAF_7 versus CAF_1–CAF_6, organized the genes into four functional modules (macrophage-lineage/MHC-II, secretome/chemokines, immune checkpoint/Treg-support, and ECM/myofibroblast), and compared their expression patterns across TAMs and CAF_1–CAF_7 using a DotPlot (Fig. 2A). Notably, CAF_7 is defined by a multi-axis transcriptional program rather than any single marker. As captured in the DotPlot (Fig. 2A), CAF_7 retained residual macrophage-lineage/MHC-II-associated features, including expression of MRC1, and showed more apparent expression of secretome-related genes (e.g., SPP1) and immune checkpoint genes (e.g., HAVCR2) relative to other CAF clusters, while also showing broad per-cell detection and coordinated co-expression of ECM/myofibroblast genes, together with lower phagolysosomal and lipid handling/cholesterol efflux features than TAMs (Figure S2A–E, Supporting Information). We next assessed lineage directionality by trajectory analyses (Monocle 2 and PAGA), which identified a trajectory consistent with a transition from TAMs toward CAF_7 (Fig. 2B, C). To experimentally validate this inferred transition, we exposed bone marrow–derived macrophages (BMDMs) to Lewis lung carcinoma–conditioned medium (LLC-CM) and quantified a representative set of CAF_7 markers spanning the four DotPlot-defined modules. LLC-CM significantly decreased macrophage markers at the mRNA level (F4/80 and CD68) and reduced CD74, consistent with attenuation of macrophage/MHC-II features during MMT, while significantly increasing CAF/myofibroblast-associated transcripts (FAP, α-SMA, and VEGF) and immunoregulatory/secretome-related transcripts (MRC1, HAVCR2, and SPP1) (Figure S2F, Supporting Information). Concordantly, Western blot showed significantly reduced F4/80, CD68, and CD74 protein levels and increased FAP, α-SMA, VEGF, MRC1, and HAVCR2 in LLC-CM–treated BMDMs (Fig. 2D), and ELISA detected significantly increased secretion of SPP1 compared with controls (Fig. 2E). Immunofluorescence staining further confirmed these molecular changes and revealed a clear morphological shift from macrophage-like to fibroblast-like cells (Fig. 2F). In vivo, macrophage depletion significantly reduced CAF_7-like cells as well as the overall α-SMA⁺ stromal compartment, supporting a TAM contribution to CAF_7 formation (Figure S2G, Supporting Information).

To further strengthen this in vivo evidence, we performed endogenous lineage-tracing experiments in our bone metastasis model using LysM-Cre; Rosa26-tdTomato mice, in which endogenous myeloid cells are permanently marked by tdTomato expression. In bone metastatic lesions, we detected tdTomato⁺CD68⁺α-SMA⁺ cells (Fig. 2G). Because α-SMA is an intracellular marker and therefore not suitable for viable cell sorting, we used surface FAP as a surrogate marker to isolate viable tdTomato⁺FAP⁺ and tdTomato⁺FAP⁻ populations from dissociated bone metastasis samples (gating in Figure S2H, Supporting Information). Compared with the tdTomato⁺FAP⁻ fraction, the tdTomato⁺FAP⁺ subset exhibited higher expression of FAP, α-SMA, and Col1a1, together with lower expression of F4/80 and CD68 (Fig. 2H). We next assessed CAF-like contractile function using a collagen gel contraction assay. tdTomato⁺FAP⁺ cells showed greater collagen-gel contraction than tdTomato⁺FAP⁻ cells (Fig. 2I). Additionally, we used a macrophage depletion/reconstitution intratibial bone metastasis framework, in which endogenous macrophages were depleted with clodronate liposomes and GFP-labeled BMDMs were co-injected into the tibia with LLC cells at a 1:1 ratio (Fig. 2J). Immunofluorescence staining of bone metastatic lesions revealed that GFP-labeled BMDMs co-expressed α-SMA (Figure S3A, Supporting Information). Using the same sorting strategy, we then isolated viable GFP⁺FAP⁺ and GFP⁺FAP⁻ populations from established bone metastatic lesions for downstream analyses (gating in Figure S3B, Supporting Information). Compared with the GFP⁺FAP⁻ fraction, GFP⁺FAP⁺ cells exhibited increased expression of FAP and α-SMA, accompanied by reduced expression of F4/80 and CD68 (Figure S3C, Supporting Information). Transcriptomic comparison further revealed attenuation of macrophage-lineage features together with coordinated enrichment of a broader ECM/myofibroblast program in GFP⁺FAP⁺ cells, including contractile genes (e.g., Acta2), fibrillar collagen genes (e.g., Col1a1), and CAF-associated stromal genes (e.g., Pdgfrb) (Fig. 2K). Consistent with these findings, GSEA further demonstrated enrichment of ECM organization pathways alongside myofibroblast-associated contractile programs in GFP⁺FAP⁺ cells (Figure S3D, Supporting Information). Moreover, GFP⁺FAP⁺ cells exhibited greater gel contraction capacity than BMDMs and GFP⁺FAP⁻ cells, consistent with acquisition of CAF-like contractile properties (Fig. 2L). Together, these results support the notion that macrophage-lineage cells can transition toward a CAF_7-like state within the bone metastatic microenvironment.

### CAF_7 is associated with Treg enrichment and CD8⁺ T cell exhaustion in lung cancer bone metastases

**Fig. 3:**
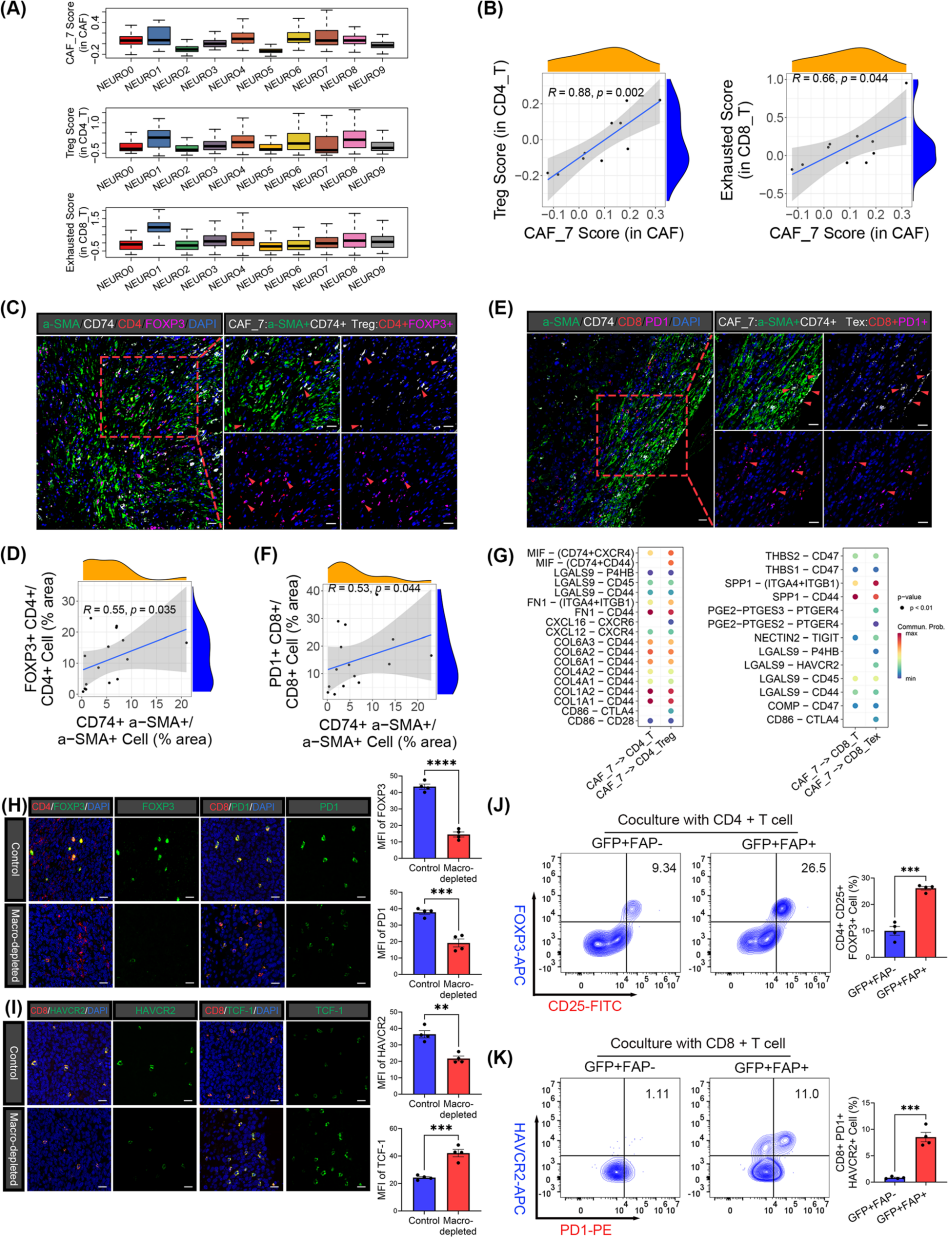
CAF_7 is associated with Treg enrichment and CD8⁺ T cell exhaustion in lung cancer bone metastases. **A**–**B **CAF_7 module scores in CAFs positively correlate with Treg signature scores in CD4⁺ T cells and exhaustion scores in CD8⁺ T cells across ten bone metastasis samples (sample IDs shown on the x-axis), as shown by boxplots and corresponding correlation analyses (*R* = 0.88, *p* = 0.002; *R* = 0.66, *p* = 0.044). **C**–**F **Immunofluorescence images of human lung cancer bone metastases (n = 15) show spatial proximity and association of CAF_7-like cells with FOXP3⁺CD4⁺ Tregs (**C**) and PD-1⁺CD8⁺ Texs (**E**). α-SMA⁺CD74⁺ cells were quantified as an additional marker combination for CAF_7-like cells, reflecting myofibroblastic and MHC class II–associated features. The proportion of α-SMA⁺CD74⁺ cells among total α-SMA⁺ CAFs correlates positively with Treg infiltration (*R* = 0.55, *p* = 0.035; D) and Tex infiltration (*R* = 0.53, *p* = 0.044; F). Scale bars, 20 μm. Colors: α-SMA, green; CD74, white; DAPI, blue; CD4/CD8, red; FOXP3/PD-1, magenta. **G **Dot plots of CellChat analysis showing predicted ligand–receptor interactions between CAF_7 and CD4⁺ or CD8⁺ T cells. Key signaling pathways suggest roles in Treg expansion (MIF–CD74/CXCR4, CXCL12–CXCR4), support of Treg suppressive function (CD86–CTLA4), and CD8⁺ T cell exhaustion (NECTIN2–TIGIT, THBS1/2–CD47, SPP1–CD44, PGE2–PTGER4). **H**–**I** Immunofluorescence analysis of bone metastases from control and macrophage-depleted mice shows reduced expression of FOXP3⁺ CD4⁺ Tregs, PD-1⁺ and HAVCR2⁺ CD8⁺ T cells, and increased TCF-1⁺ CD8⁺ T cells upon macrophage depletion. Quantification of MFI indicates significant reductions in FOXP3, PD-1, and HAVCR2, with a concomitant increase in TCF-1 expression (*n* = 4, **p < 0.01, ***p < 0.001, ****p < 0.0001). Scale bars, 20 μm. Colors: CD4/CD8 red; FOXP3/PD-1/HAVCR2/TCF-1 green; DAPI blue. **J**–**K** Co-culture assays using freshly isolated GFP⁺FAP⁺ and GFP⁺FAP⁻ populations from dissociated bone metastatic lesions. The two fractions were separately co-cultured with freshly isolated CD4⁺ T cells (**J**) or CD8⁺ T cells (**K**), followed by flow-cytometric assessment of T-cell phenotypes. Relative to the GFP⁺FAP⁻ fraction, GFP⁺FAP⁺ cells more strongly promoted CD25⁺FOXP3⁺ Treg differentiation in CD4⁺ T cells and increased the PD-1⁺HAVCR2⁺ exhausted phenotype in CD8⁺ T cells (*n* = 4, ***p < 0.001)

To explore the role of CAF_7 in promoting immunosuppression in lung cancer bone metastases, we conducted a comprehensive multimodal analysis. Module score profiling across ten bone metastatic samples revealed strong correlations between CAF_7 signatures in CAFs and both the CD4⁺ Treg signature (*R* = 0.88, *p* = 0.002) and the CD8⁺ T cell exhaustion signature (*R* = 0.66, *p* = 0.044) (Fig. 3A, B). Immunofluorescence in human lung cancer bone metastasis samples confirmed that the proportion of CAF_7-like cells (α-SMA⁺CD74⁺) among total α-SMA⁺ CAFs was positively correlated with FOXP3⁺CD4⁺ Treg infiltration (*R* = 0.55, *p* = 0.035) and PD-1⁺CD8⁺ T cell frequency (*R* = 0.53, *p* = 0.044) (Fig. 3C–F). CellChat analysis revealed strong communication between CAF_7 and CD4⁺/CD8⁺ T cells (Figure S4A, Supporting Information). Ligand–receptor inference suggested that CAF_7 may promote Treg recruitment and differentiation through multiple mechanisms, including CAF_7-secreted MIF binding to CD74/CXCR4 on CD4⁺ T cells to drive their conversion into Tregs, CXCL12–CXCR4 signaling to recruit Tregs, and CD86–CTLA4 interaction supporting Treg suppressive function [40–43]. In parallel, CAF_7 may induce CD8⁺ T cell exhaustion via NECTIN2–TIGIT, THBS1/2–CD47, SPP1–CD44, and PGE2–PTGER4 axes (Fig. 3G) [44–49]. In macrophage-depleted models, FOXP3 expression in CD4⁺ T cells and PD-1/HAVCR2 in CD8⁺ T cells were reduced, while TCF-1 was upregulated, indicating attenuated immunosuppression and restoration of stem-like features in CD8⁺ T cells (Fig. 3H, I).

To further determine whether CAF_7-like cells can directly modulate T-cell states, the GFP⁺FAP⁺ and GFP⁺FAP⁻ populations identified above were freshly isolated from dissociated bone metastatic lesions and separately co-cultured with freshly isolated CD4⁺ or CD8⁺ T cells, followed by flow-cytometric analysis of T-cell phenotypes. Compared with the GFP⁺FAP⁻ fraction, the GFP⁺FAP⁺ fraction more strongly promoted FOXP3⁺ Treg differentiation from CD4⁺ T cells and increased CD8⁺ T-cell exhaustion, as indicated by a higher frequency of PD-1⁺HAVCR2⁺ CD8⁺ T cells (Fig. 3J, K). Together, these findings further support the idea that CAF_7-like cells can promote immunosuppressive T-cell states.

### ASCL2 is a key transcription factor mediating MMT and immunosuppressive CAF_7 identity

**Fig. 4:**
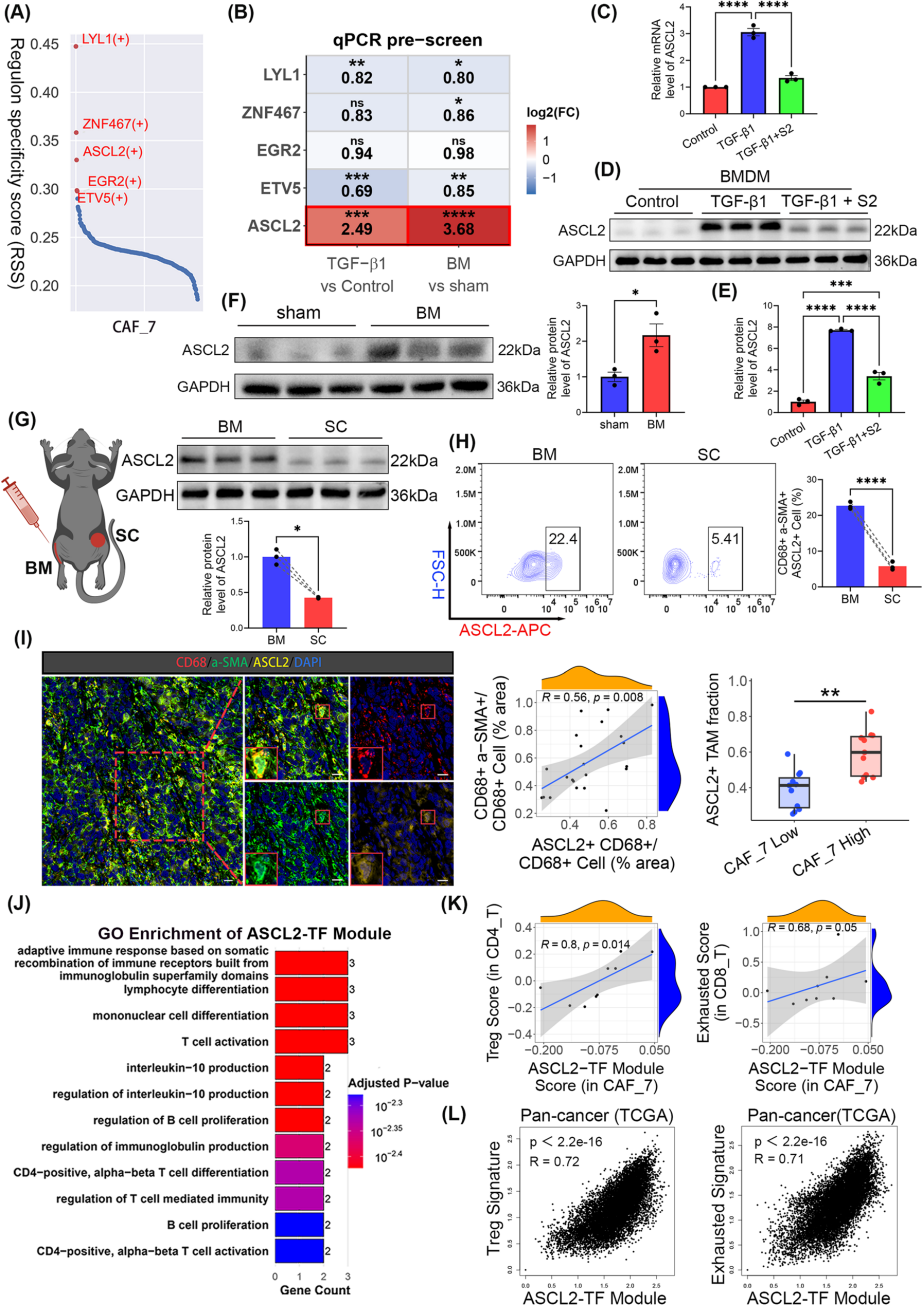
ASCL2 is a key transcription factor mediating MMT and immunosuppressive CAF_7 identity. **A **SCENIC regulon specificity score (RSS) ranking of transcription factor (TF) regulons in CAF_7 cells. Each dot represents one TF regulon (TF plus its inferred target gene set). The y-axis indicates RSS, where higher values denote greater selective specificity in CAF_7 relative to other cell states in the dataset. The top CAF_7-associated regulons are labeled (ASCL2, LYL1, ZNF467, EGR2, and ETV5). “(+)” denotes positively activated regulons as defined by the SCENIC output. **B **qPCR pre-screen of SCENIC-nominated TFs across two predefined settings (TGF-β1–induced MMT model: TGF-β1 vs. control; and in vivo lesions: BM vs. sham). For the in vivo setting, BM samples were collected from the tumor-bearing tibial lesion region at the experimental endpoint, whereas sham-operated tibial controls underwent identical anesthesia and tibial cortical entry followed by intramedullary PBS injection. Tile color indicates log2(FC), and numbers indicate fold-change (FC); ASCL2 shows the strongest induction across both settings (*n* = 3, *p < 0.05, **p < 0.01, ***p < 0.001, ****p < 0.0001). **C**–**E** In BMDMs, qPCR and Western blot show that TGF-β1 robustly induces ASCL2 at mRNA and protein levels, whereas the TGF-βRI/ALK5 inhibitor SB431542 (labeled “S2”) attenuates this induction; representative blots and quantifications are shown (*n* = 3, ***p < 0.001, ****p < 0.0001). **F **In vivo, ASCL2 protein levels are higher in intratibial lung cancer bone-metastasis lesions (BM) than in sham-operated tibial controls: Western blot with densitometry (GAPDH-normalized; *n* = 3; unpaired two-tailed t-test, *p < 0.05). **G **Site comparison shows higher ASCL2 protein levels in intratibial bone lesions (BM) versus subcutaneous tumors (SC); schematic (left) and Western blot with quantification (right) (*n* = 3, *p < 0.05). **H** Flow cytometry demonstrates a larger fraction of ASCL2⁺ CAF_7-like cells (CD68⁺α-SMA⁺ASCL2⁺) in BM versus SC tumors (*n* = 3, ****p < 0.0001). **I** Multiplex immunofluorescence of human NSCLC bone metastasis specimens with quantitative image analysis reveals a significant positive association between the ASCL2⁺ TAM fraction (ASCL2⁺CD68⁺/CD68⁺) and the CAF_7-like fraction (CD68⁺α-SMA⁺/CD68⁺) across the cohort (*n* = 22, *R* = 0.56, *p* = 0.008; middle). A patient-level median-split analysis stratified by the CAF_7-like fraction further shows higher ASCL2⁺ TAM fractions in the CAF_7-like-high group than in the CAF_7-like-low group (right) (*n* = 22, 11 per group, **p < 0.01, Mann–Whitney U). Fractions were quantified as % area. Scale bars, 20 μm. Colors: CD68 (red), α-SMA (green), ASCL2 (yellow), DAPI (blue). **J** Gene Ontology enrichment analysis of ASCL2-TF module genes revealed predominant enrichment in immune-related biological processes, including lymphocyte and mononuclear cell differentiation and T cell–mediated immunity. **K** ASCL2-TF module scores in CAF_7 cells positively correlated with CD4⁺ Treg signatures (*R* = 0.8, *p* = 0.014) and CD8⁺ T cell exhaustion signatures (*R* = 0.68, *p* = 0.05) across lung cancer bone metastases. **L** These associations were supported by pan-cancer TCGA data, which showed strong correlations between ASCL2-TF module expression and both Treg (*R* = 0.72, *p* < 2.2e-16) and exhausted T cell signatures (*R* = 0.71, *p* < 2.2e-16)

To identify putative transcriptional regulators of MMT, we applied SCENIC regulon analysis and ranked transcription factor (TF) regulons by their regulon specificity score (RSS), which quantifies how selectively each regulon is enriched in CAF_7 relative to other cell states. This analysis highlighted ASCL2, LYL1, ZNF467, EGR2, and ETV5 as top CAF_7–associated regulons (Fig. 4A).

To prioritize these candidates for downstream studies, we conducted a qPCR pre-screen primarily in a defined TGF-β1–induced MMT model, as the compositional complexity of LLC-CM limits inhibitor-controlled, pathway-attributable inference [15, 19, 22]. In this screen, ASCL2 showed the largest fold-change among the SCENIC-nominated TFs; a directionally concordant upregulation was also observed in vivo in murine intratibial bone-metastasis lesions (BM) compared with sham marrow. In contrast, the other candidates generally exhibited smaller-magnitude changes or were not consistently significant across the two settings (Fig. 4B; Figure S5A, Supporting Information). Accordingly, we prioritized ASCL2 for subsequent validation and pathway interrogation.

In the TGF-β1–induced MMT model, qPCR and Western blot confirmed increased ASCL2 expression at both the mRNA and protein levels relative to controls (Fig. 4C–E). Moreover, pharmacologic inhibition of TGF-β receptor I (TGF-βRI/ALK5) with SB431542 attenuated the TGF-β1–driven increase in ASCL2, supporting a contributory role of TGF-β signaling in ASCL2 induction (Fig. 4C–E). In vivo, ASCL2 protein levels were elevated in murine lung cancer bone-metastasis specimens relative to sham tibial marrow (Fig. 4F). We next asked whether the bone metastatic niche is associated with heightened ASCL2 activity by comparing intratibial bone lesions (BM) with subcutaneous (SC) tumors (Fig. 4G). Western blot analysis confirmed higher ASCL2 protein abundance in bone lesions (Fig. 4G), and flow cytometry revealed an increased proportion of ASCL2⁺ CAF_7-like cells (α-SMA⁺CD68⁺ASCL2⁺) within bone metastases (Fig. 4H; gating in Figure S7B, Supporting Information). Collectively, these findings indicate preferential enrichment of ASCL2⁺ CAF_7-like cells in the bone metastatic microenvironment.

To connect our experimental findings to human disease, we examined human NSCLC bone metastasis specimens using multiplex immunofluorescence coupled with quantitative image analysis. Across the cohort (*n* = 22), the fraction of ASCL2⁺ TAMs (ASCL2⁺CD68⁺/CD68⁺) showed a significant positive association with the CAF_7-like cell fraction (CD68⁺α-SMA⁺/CD68⁺) (*R* = 0.56, *p* = 0.008; Fig. 4I). Consistently, a patient-level median-split analysis based on the CAF_7-like cell fraction revealed higher ASCL2⁺ TAM fractions in CAF_7-like–high cases than in CAF_7-like–low cases (Fig. 4I). Together, these cohort-level protein data provide human evidence supporting the ASCL2–MMT–CAF_7-like axis in the bone metastatic microenvironment.

We next extended these observations to larger patient cohorts using a pan-cancer analysis of The Cancer Genome Atlas (TCGA). The ASCL2–TF module (ASCL2 and its target genes; Figure S5B, Supporting Information) showed a modest positive association with CAF signatures (*R* = 0.21, *p* < 2.2e-16) (Figure S5C, Supporting Information), consistent with a link between ASCL2-associated transcriptional programs and CAF-related features. Gene Ontology enrichment further indicated that this module is overrepresented in immune-regulatory programs, including mononuclear cell differentiation, lymphocyte differentiation, and regulation of T cell–mediated immunity (Fig. 4J). In our bone metastasis scRNA-seq dataset, ASCL2–TF module scores in CAF_7 cells were strongly correlated with CD4⁺ T cell Treg signatures (*R* = 0.8, *p* = 0.014) and CD8⁺ T cell exhaustion signatures (*R* = 0.68, *p* = 0.05) (Fig. 4K). These relationships were recapitulated across TCGA cancer types, spanning all available TCGA cohorts (*n* = 33), where the ASCL2–TF module correlated positively with both Treg (*R* = 0.72, *p* < 2.2e-16) and exhausted T cell signatures (*R* = 0.71, *p* < 2.2e-16) (Fig. 4L), consistent with the immunosuppressive phenotype of MMT-derived CAF_7 cells. Finally, TCGA survival analyses across multiple cancer types indicated that higher ASCL2 expression tends to be associated with poorer prognosis in several cohorts (Figure S5D, Supporting Information).

### ASCL2 knockdown suppresses MMT and alleviates MMT-associated immunosuppression in vitro

**Fig. 5:**
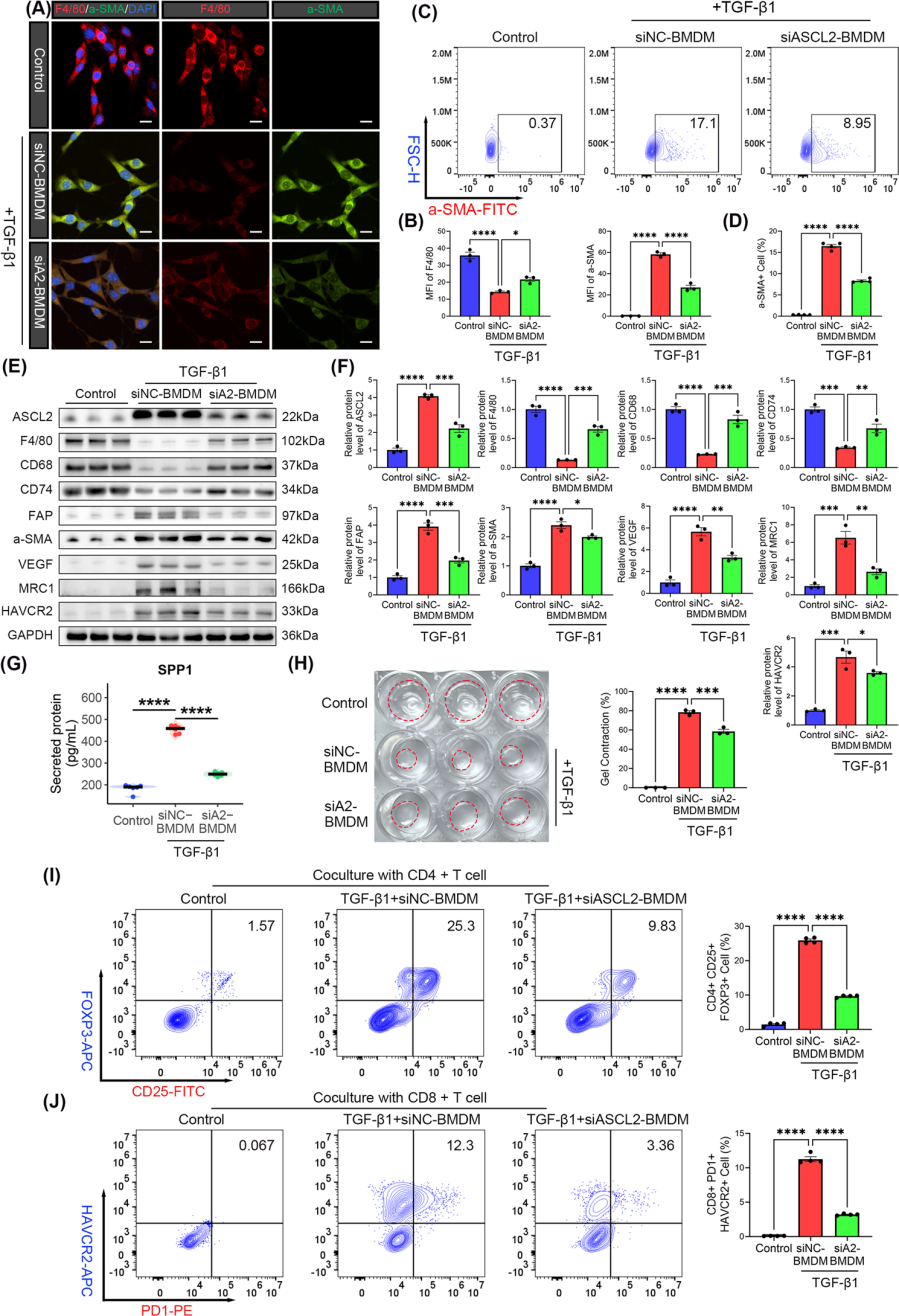
ASCL2 knockdown suppresses MMT and alleviates MMT-associated immunosuppression in vitro. **A**, **B** ASCL2 (labeled “A2”) knockdown in BMDMs mitigated TGF-β1–induced CAF-like transition, as shown by reduced α-SMA and increased F4/80 expression (*n* = 3, *p < 0.05, ****p < 0.0001). Scale bars, 20 μm. Colors: F4/80 red; α-SMA green; DAPI blue. **C**–**D** Flow cytometry confirmed a significant reduction in the proportion of α-SMA⁺ BMDMs upon ASCL2 knockdown under TGF-β1 stimulation (*n* = 4, ****p < 0.0001). **E**–**F** Western blot and densitometric quantification of BMDMs under TGF-β1 showing ASCL2 knockdown efficiency and associated marker changes: FAP, α-SMA, VEGF, MRC1, and HAVCR2 decreased, whereas F4/80, CD68, and CD74 increased in siASCL2-BMDMs relative to siNC-BMDMs (*n* = 3, *p < 0.05, **p < 0.01, ***p < 0.001, ****p < 0.0001). **G** ELISA of conditioned media from BMDMs under TGF-β1 showing increased secretion of SPP1 in siNC-BMDMs, which was significantly reduced by ASCL2 knockdown (*n* = 6, ****p < 0.0001). **H** Collagen gel contraction assay showing enhanced contraction in TGF-β1-treated BMDMs and attenuation of this response by ASCL2 knockdown (*n* = 3, ***p < 0.001, ****p < 0.0001). **I** In the CD4⁺ T-cell co-culture assay, ASCL2-deficient BMDMs showed a reduced ability to induce CD25⁺FOXP3⁺ Treg cells (*n* = 4, ****p < 0.0001). **J** In the CD8⁺ T-cell co-culture assay, ASCL2 knockdown in BMDMs reduced the frequency of PD-1⁺HAVCR2⁺ CD8⁺ T cells (*n* = 4, ****p < 0.0001)

We next defined the functional role of ASCL2 in MMT by testing whether macrophage-directed ASCL2 inhibition suppresses MMT in vitro. ASCL2 knockdown in BMDMs effectively blunted TGF-β1–driven MMT, as evidenced by attenuated CAF-like morphology (Fig. 5A; Figure S6A, Supporting Information), reduced α-SMA expression, and increased expression of the macrophage marker F4/80 (Fig. 5A, B). ASCL2 depletion also significantly curtailed the TGF-β1–induced expansion of α-SMA⁺ cells (Fig. 5C, D; gating in Figure S7A, Supporting Information).

In this defined TGF-β1–driven MMT setting, CAF_7-relevant readouts spanning macrophage-lineage/MHC-II traces, ECM/myofibroblast remodeling, secretome output, and immune checkpoint–linked features showed broadly concordant trends with those observed in the LLC-CM context, and ASCL2 knockdown consistently reversed these molecular changes. Specifically, Western blot analyses confirmed efficient ASCL2 knockdown and revealed coordinated remodeling of lineage and immune modulation–related markers: compared with TGF-β1–treated control (siNC) cells, ASCL2 knockdown increased macrophage-associated proteins (F4/80 and CD68) and CD74 levels, while reducing myofibroblast/CAF-associated proteins (FAP, α-SMA, and VEGF) (Fig. 5E, F). Notably, ASCL2 knockdown also attenuated the TGF-β1–induced upregulation of the immune modulation–related proteins MRC1 and HAVCR2 (Fig. 5E, F). To strengthen specificity, we performed an ASCL2 rescue experiment in the same TGF-β1–induced MMT setting and found that re-expression of an RNAi-resistant ASCL2 construct (ASCL2^res; “A2-res”) restored ASCL2 protein levels and largely reversed the siASCL2-driven marker changes (F4/80/CD68, FAP/α-SMA/VEGF, and MRC1/HAVCR2) (Figure S6B, Supporting Information). Concordantly, ELISA showed that TGF-β1 robustly increased SPP1 secretion in the conditioned medium, whereas ASCL2 knockdown significantly reduced SPP1 levels (Fig. 5G). Functionally, we performed a collagen gel contraction assay. Relative to untreated control BMDMs, TGF-β1-treated BMDMs exhibited markedly enhanced collagen gel contraction, whereas ASCL2 knockdown significantly blunted this contractile response (Fig. 5H).

Functional co-culture assays further demonstrated that ASCL2 knockdown reversed MMT-associated immunosuppression by suppressing CD4⁺ T cell differentiation into Treg (Fig. 5I; gating in Figure S7C, Supporting Information) and ameliorating CD8⁺ T cell exhaustion (Fig. 5J; gating in Figure S7D, Supporting Information). Collectively, these data indicate that ASCL2 inhibition dampens the MMT program and concomitantly reduces MMT-linked immunoregulatory outputs in vitro, supporting the mechanistic rationale for combining ASCL2 targeting with immunotherapy.

### ASCL2 knockdown in macrophages suppresses MMT and tumor progression in vivo

**Fig. 6:**
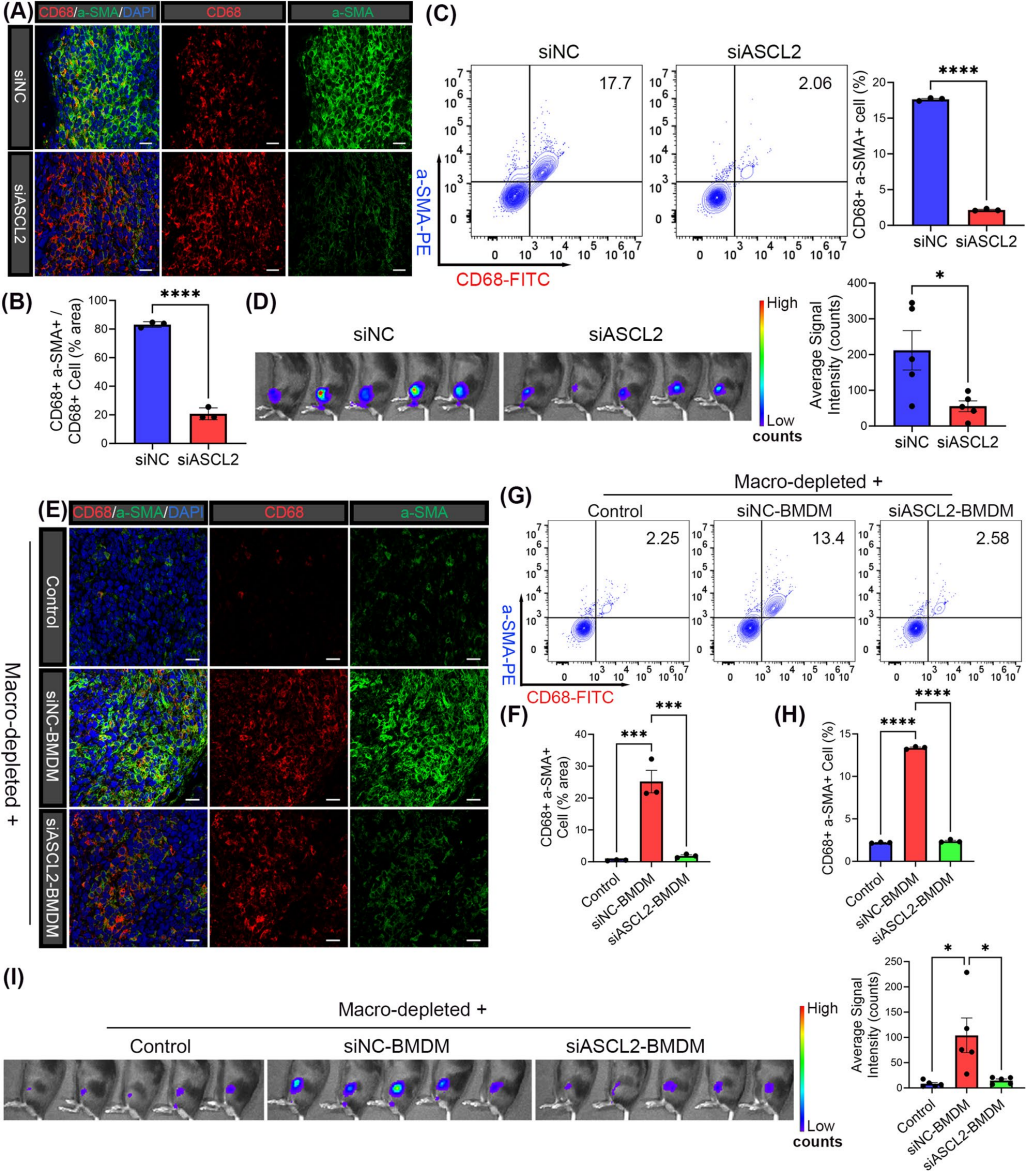
ASCL2 knockdown in macrophages suppresses MMT and tumor progression in vivo. **A**–**C **Immunofluorescence (**A**–**B**) and flow cytometry (**C**) analyses of bone metastatic lesions demonstrate a marked reduction in CAF_7-like cells (CD68⁺α-SMA⁺) following local peritumoral delivery of siASCL2 versus siNC (n = 3, ****p < 0.0001). Scale bars, 20 μm. Colors: CD68 red; α-SMA green; DAPI blue. Related flow cytometry gating strategies can be found in Figure S7. **D** In vivo bioluminescence imaging shows significantly decreased tumor burden in siASCL2-treated mice, as reflected by reduced signal intensity (n = 5, *p < 0.05). **E**–**F** In a macrophage adoptive transfer model, macrophage-depleted mice were co-injected with LLC cells and BMDMs transfected with either siNC or siASCL2. Immunofluorescence revealed a substantial reduction in intratumoral CAF_7-like cells in the siASCL2-BMDM group (n = 3, ***p < 0.001). Scale bars, 20 μm. Colors: CD68 red; α-SMA green; DAPI blue. **G**–**H** Flow cytometry confirmed a decrease in CAF_7-like cell frequency following siASCL2-BMDM transfer (n = 3, ****p < 0.0001). **I** Bioluminescence imaging and signal quantification confirm that siASCL2-BMDMs substantially suppressed tumor growth compared to siNC-BMDMs (n = 5, *p < 0.05).

To elucidate the role of ASCL2 in MMT within the tumor microenvironment of lung cancer bone metastases, we established a bone metastasis model and performed local peritumoral injections of either control siRNA (siNC) or ASCL2-targeting siRNA (siASCL2) following tumor establishment. Immunofluorescence (Fig. 6A, B) and flow cytometry (Fig. 6C) showed that ASCL2 depletion significantly reduced the in vivo frequency of CAF_7-like cells, and bioluminescence imaging indicated attenuated tumor progression (Fig. 6D), identifying ASCL2 as a critical regulator of MMT-linked CAF_7-like formation. To assess the macrophage-intrinsic role of ASCL2 in vivo, we next employed the macrophage depletion/reconstitution intratibial bone metastasis model, in which macrophage-depleted mice were co-injected with LLC cells plus either siASCL2- or siNC-transfected BMDMs, while control mice received LLC cells plus PBS. Compared with siNC-BMDMs, siASCL2-BMDMs markedly suppressed CAF_7-like cell accumulation (Fig. 6E–H) and tumor growth (Fig. 6I), demonstrating that macrophage-specific ASCL2 is essential for CAF_7-like cell–associated oncogenic progression.

### Functional validation of the ASCL2–Il6ra axis in MMT-linked immunostromal remodeling and tumor progression

**Fig. 7:**
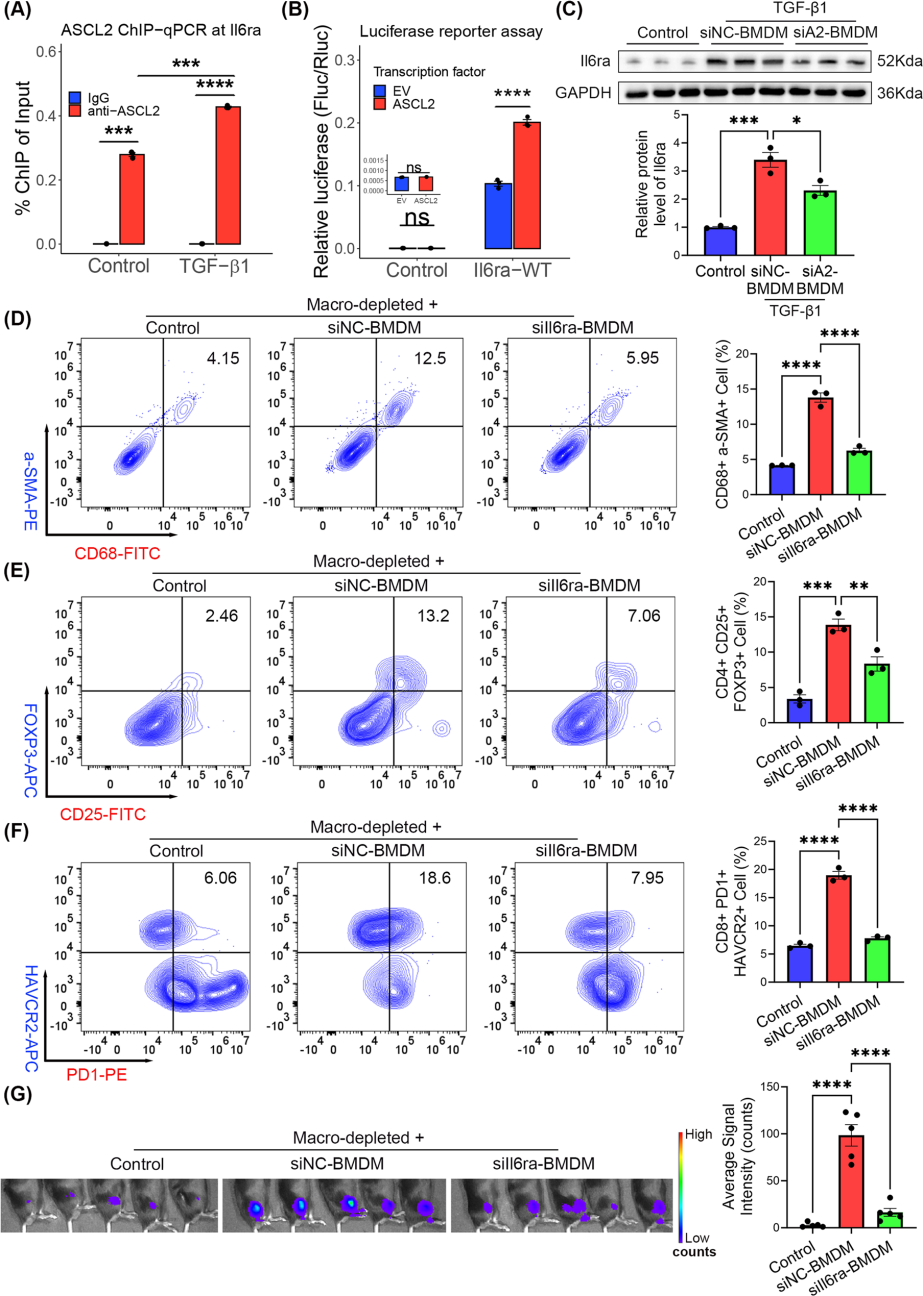
Functional validation of the ASCL2–Il6ra axis in MMT-linked immunostromal remodeling and tumor progression. **A** ChIP–qPCR showing specific enrichment of the Il6ra locus in anti-ASCL2 immunoprecipitates relative to IgG, with higher occupancy upon TGF-β1 stimulation (*n* = 3, ***p < 0.001, ****p < 0.0001). **B** Luciferase reporter assay demonstrating that ASCL2 increases Il6ra–WT promoter activity versus empty vector (EV), with no detectable change in the control reporter (*n* = 3, ****p < 0.0001; ns, not significant). **C** Western blot and densitometric quantification show Il6ra protein changes in BMDMs, where Control cells were unstimulated (no TGF-β1) and the siNC-BMDM and siASCL2-BMDM (siA2-BMDM) groups were treated with TGF-β1: Il6ra was induced in TGF-β1–treated siNC-BMDMs relative to unstimulated Control, and this induction was attenuated by ASCL2 knockdown (*n* = 3, *p < 0.05, ***p < 0.001). **D**–**F** Using the macrophage depletion–reconstitution bone metastasis framework, flow cytometry indicates that siNC-BMDM reconstitution increases CAF_7-like cells as well as CD4⁺CD25⁺FOXP3⁺ Tregs and PD-1⁺HAVCR2⁺ exhausted CD8⁺ T cells, whereas Il6ra knockdown in transferred BMDMs attenuates these phenotypes (*n* = 3, **p < 0.01, ***p < 0.001, ****p < 0.0001). Related flow cytometry gating strategies can be found in Figure S7. **G** Bioluminescence imaging shows reduced tumor burden in mice receiving siIl6ra-BMDMs compared with siNC-BMDMs (*n* = 5, ****p < 0.0001)

Given that CAF_7 expansion is associated with Treg enrichment and CD8⁺ T-cell exhaustion, and that macrophage ASCL2 is required for MMT-associated CAF_7-like formation in vivo, we next sought downstream effectors that could mechanistically link ASCL2 to immunostromal remodeling. Guided by SCENIC regulon analysis to identify downstream effectors of the ASCL2-driven CAF_7 program (Figure S5B, Supporting Information), we prioritized IL6R (mouse Il6ra) for targeted testing as it was nominated as a putative ASCL2 target, and given that IL-6/IL6R signaling has been widely implicated in tumor-microenvironment immune regulation and stromal remodeling [50–56]. ChIP–qPCR demonstrated ASCL2 occupancy at the Il6ra promoter locus, with robust enrichment over IgG and increased binding upon TGF-β1 stimulation, and ASCL2 selectively enhanced Il6ra–WT promoter reporter activity without affecting the control reporter, supporting direct transcriptional regulation of Il6ra by ASCL2 (Fig. 7A, B). Moreover, Western blotting showed that Il6ra protein was induced in TGF-β1–treated BMDMs and that ASCL2 knockdown attenuated this induction compared with siNC controls (Fig. 7C). Extending these findings in vivo using the macrophage depletion–reconstitution bone metastasis framework, siNC-BMDM transfer increased CAF_7-like cells and was accompanied by higher CD4⁺CD25⁺FOXP3⁺ Treg frequencies and expanded PD-1⁺HAVCR2⁺ exhausted CD8⁺ T cells, whereas Il6ra knockdown in transferred BMDMs attenuated these phenotypes (Fig. 7D–F). Concordantly, bioluminescence imaging indicated reduced tumor burden upon macrophage Il6ra knockdown relative to the siNC-BMDM group (Fig. 7G).

### ASCL2 targeting in macrophages enhances antitumor immunity and improves the efficacy of PD-1 blockade

**Fig. 8:**
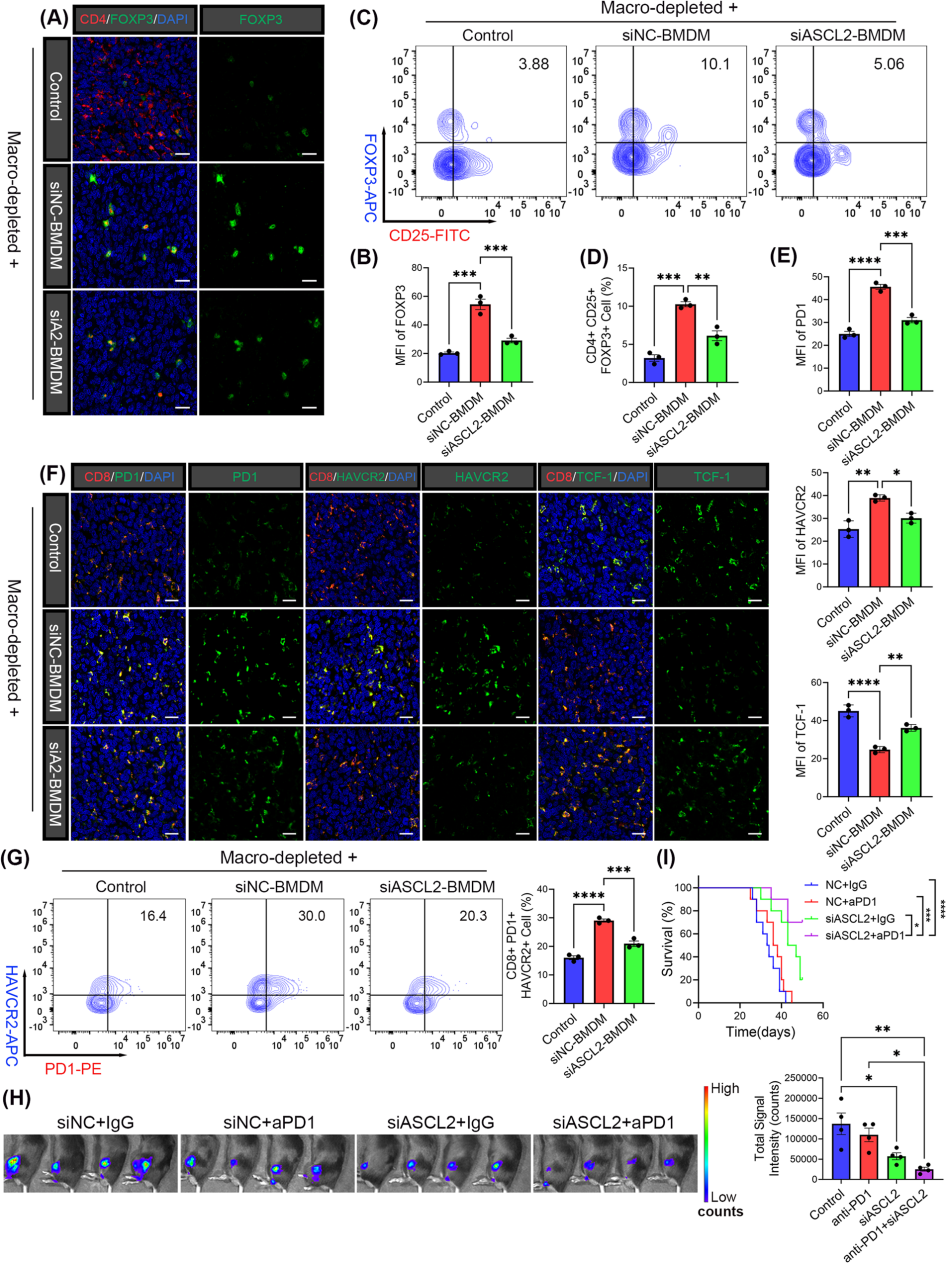
ASCL2 targeting in macrophages enhances antitumor immunity and improves the efficacy of PD-1 blockade. **A**-**B** Immunofluorescence analysis reveals that the siASCL2-BMDM group exhibits reduced FOXP3 expression compared to the siNC-BMDM group (n = 3, ***p < 0.001). Scale bars, 20 μm. Colors: CD4 red; FOXP3 green; DAPI blue. **C**-**D** Flow cytometry confirms a lower frequency of Tregs (CD4⁺CD25⁺FOXP3⁺) in the siASCL2-BMDM group relative to the siNC-BMDM group (n = 3, **p < 0.01, ***p < 0.001). Related flow cytometry gating strategies can be found in Figure S7. **E**-**F** CD8⁺ T cells from the siASCL2-BMDM group show reduced expression of exhaustion markers (PD-1, HAVCR2) and elevated TCF-1 expression, indicative of enhanced stem-like properties (n = 3, **p < 0.01, ***p < 0.001, ****p < 0.0001). Scale bars, 20 μm. Colors: CD8 red; PD-1/HAVCR2/TCF-1 green; DAPI blue. **G** Flow cytometry further reveals a reduced proportion of PD-1⁺HAVCR2⁺ exhausted CD8⁺ T cells in the siASCL2-BMDM group versus the siNC-BMDM group (n = 3, ***p < 0.001, ****p < 0.0001). **H** Bioluminescence imaging shows that siASCL2 monotherapy significantly reduces tumor burden compared with control, and the combination with PD-1 blockade further decreases tumor burden relative to PD-1 blockade alone (n = 4, *p < 0.05, **p < 0.01). **I** Kaplan–Meier survival analysis reveals that siASCL2 treatment in combination with PD-1 blockade significantly extends survival compared to either monotherapy or control groups (n = 10 per group, *p < 0.05, ***p < 0.001, ****p < 0.0001)

Building on the ASCL2–Il6ra mechanistic link in MMT-associated immunostromal remodeling, we asked whether macrophage-specific ASCL2 inhibition improves intralesional T-cell states and therapeutic benefit in vivo. Accordingly, we analyzed T-cell responses in the same macrophage depletion–reconstitution intratibial bone-metastasis model described above. Compared to siNC-BMDM recipients, siASCL2-BMDM-treated mice displayed significantly lower FOXP3 expression (Fig. 8A, B) and reduced Treg frequency among CD4⁺ T cells (Fig. 8C, D), suggesting diminished immunosuppression. Additionally, CD8⁺ T cells from siASCL2-BMDM recipients exhibited attenuated exhaustion markers (reduced PD-1 and HAVCR2) and enhanced stem-like properties (elevated TCF-1; Fig. 8E, F), accompanied by a decline in PD-1⁺HAVCR2⁺ CD8⁺ T cells (Fig. 8G). These results provide a mechanistic basis for combining ASCL2 inhibition with PD-1 blockade.

To assess the therapeutic relevance of these immune changes, peritumoral siASCL2 was administered alongside intraperitoneal anti-PD-1 antibody. While siASCL2 monotherapy (siASCL2 + IgG) significantly suppressed tumor growth versus controls (siNC + IgG), PD-1 blockade alone (siNC + αPD-1) exerted a limited effect that did not reach statistical significance. Notably, the combination (siASCL2 + αPD-1) significantly reduced tumor burden relative to αPD-1 alone and further improved survival compared with either monotherapy (Fig. 8H, I), supporting that ASCL2 silencing potentiates PD-1 blockade.

## Discussion

This study reveals a novel mechanism of stromal evolution in metastatic NSCLC: bone metastases co-opt macrophages via MMT to generate immunosuppressive CAFs. Using single-cell RNA sequencing, we identified a distinct CAF subset (“CAF_7”) co-expressing macrophage, MHC class II, and fibroblast programs that becomes increasingly enriched from primary tumors to bone metastases. CAF_7 expansion coincides with a Treg-high, CD8⁺ T cell exhaustion–skewed immune state, and functional perturbation shows that macrophage-intrinsic ASCL2 is important for efficient MMT and CAF_7-like formation in vivo. Mechanistically, we connect ASCL2 to a downstream effector by demonstrating transcriptional activation and locus occupancy at Il6ra/IL6R and by showing that macrophage Il6ra knockdown recapitulates key immunostromal and tumor-control effects. Together, these findings define an underappreciated lineage plasticity program that shapes immune–stromal remodeling in bone metastases, establish ASCL2-driven MMT as a key mechanism contributing to immune evasion, and nominate ASCL2–Il6ra/IL6R as a potentially tractable axis for therapeutic reprogramming.

Our work both builds on and extends emerging insights into CAF heterogeneity and origins. Tang et al. demonstrated that TAMs can convert into CAF-like cells in primary NSCLC, and that blocking TGF-β/SMAD3 signaling can prevent this transition [15]. In line with these findings, we show that macrophages in the bone metastatic niche likewise undergo MMT toward an α-SMA⁺ fibroblast-like state, supported by our pseudotime trajectory analyses together with complementary in vitro and in vivo experiments. Crucially, our study identifies ASCL2 as a key regulator of this process in bone metastases, a role not previously linked to MMT. Rather than displacing the TGF-β/SMAD3-centered framework previously reported in primary tumors [15], our findings extend that framework by indicating that canonical TGF-β signaling remains contributory, but is not sufficient on its own to fully explain ASCL2 induction in the bone metastatic setting. In our TGF-β1–induced in vitro MMT model, pharmacologic inhibition of TGF-βRI/ALK5 with SB431542 attenuated—but did not abolish—TGF-β1-induced ASCL2 upregulation, indicating partial dependence on TGF-β signaling and supporting the involvement of additional parallel or cooperative pathways. Thus, ASCL2 is more consistent with a downstream regulatory node whose induction is partly supported by TGF-β signaling and likely reinforced by other microenvironment-derived signals. In the context of bone metastasis, this model is biologically plausible, as the bone metastatic niche is rich in matrix-derived TGF-β, including TGF-β released during osteolytic remodeling of the bone matrix [57, 58], and is also shaped by Wnt-related signaling, which has been implicated in extracellular matrix remodeling, immune tolerance, and the establishment of a bone metastatic microenvironment that supports tumor growth within bone [59, 60]. This is particularly relevant because ASCL2 itself is also a well-established Wnt-responsive transcription factor [61, 62]. Collectively, these observations support the possibility that bone-resident cues—including Wnt-related and other niche-derived factors—may act together with profibrotic TGF-β to reinforce ASCL2 expression and thereby promote MMT in infiltrating macrophages. Consistent with this framework, ASCL2 levels were higher in metastatic bone lesions than in subcutaneous tumors, and ASCL2⁺ CAF_7-like cells were enriched within the bone metastatic niche, supporting preferential activation of the ASCL2-associated MMT program in bone.

The immunological impacts of CAF_7 also align with and inform known characteristics of bone metastasis. CAF_7 cells co-express MHC class II molecules (e.g., HLA-DRA) and express immunomodulatory mediators (e.g., SPP1) that may modulate T cell function. These features are consistent with the recently described antigen-presenting CAFs (apCAFs) in certain tumors [20]. In fact, Xu et al. (2024) identified a CAF subset expressing MHC class II molecules that was notably enriched in the primary tumors of NSCLC patients already presenting with bone metastases. This population may play a key role in promoting bone metastatic colonization by engaging stemness-associated signaling pathways, such as SPP1–CD44 and SPP1–PTGER4 [63]. We similarly found that CAF_7 engages in SPP1–CD44 signaling and other ligand–receptor interactions that expand Tregs and inhibit effector T cells. Functionally, our data suggest that these macrophage-derived CAFs actively sculpt an immune-tolerant niche: higher CAF_7 levels correlated with increased FOXP3⁺ Tregs and exhausted PD-1⁺ CD8⁺ T cells in vivo. Given evidence that the IL-6/STAT3 axis sustains Treg programs and promotes PD-1–associated dysfunction in CD8⁺ T cells [50, 64], we posit that an ASCL2–IL6R axis within MMT-derived CAF_7 could amplify IL-6 signaling and—via paracrine or soluble-IL-6R–mediated trans-signaling—foster STAT3-dependent immunosuppression in bone [65]. This mechanism is particularly plausible in the bone marrow, an immunoprivileged site characterized by an abundance of suppressive cells; [66] for instance, regulatory T cells constitute a disproportionately large fraction of lymphocytes in the marrow [3]. In prostate cancer bone metastases, Tregs accumulate and create an immunosuppressive niche that facilitates tumor growth [67]. Our findings extend these observations by indicating that tumor-educated macrophages (via MMT) are a driving force establishing that niche in NSCLC metastases. In essence, lung cancer cells colonizing the bone co-opt arriving macrophages, converting them into Treg-promoting CAFs that reinforce immune evasion. Consistent with this immunosuppressive role, we found that elevated CAF_7 levels portend worse survival across multiple cancer types, underscoring the clinical relevance of this subset as a pan-cancer mediator of immune escape.

At the same time, CAF_7 also shares features with previously described apCAF and SPP1⁺ macrophage states. CAF_7 shares with antigen-presenting CAFs (apCAFs) an MHC class II antigen-presentation module. In the original description, apCAFs were defined as MHC class II–high fibroblasts with limited or absent canonical co-stimulatory molecules [68]. Subsequent studies further refined the current understanding of apCAF origin and function. In pancreatic cancer, apCAFs were shown to arise from mesothelial cells through a mesothelial-to-fibroblastic transition and to promote regulatory T-cell expansion [20], whereas in lung tumors, apCAFs were reported to support local CD4⁺ T-cell immunity and anti-tumor immune control [69]. More recently, a cross-organ spatial single-cell study spanning human tumors and associated normal tissues from 13 organs further highlighted heterogeneity within apCAF populations, including mesothelial-like apCAFs and fibrocyte-like apCAFs [70]. By contrast, CAF_7 in our study is supported as an MMT-associated stromal population arising from TAMs, indicating a lineage context distinct from the apCAF populations described to date. Functionally, CAF_7 is associated in our model with a Treg-enriched niche marked by CD8⁺ T-cell dysfunction, supporting an overall immunosuppressive interpretation and adverse clinical association, whereas apCAF states appear to exhibit more context-dependent immune functions across tumor types. Taken together, these observations suggest that, despite sharing an MHC class II antigen-presentation module, CAF_7 remains distinct from currently described apCAFs in both lineage context and immunologic function. CAF_7 also shows partial overlap with SPP1⁺ macrophage states through shared SPP1 expression. However, SPP1⁺ macrophages remain fundamentally myeloid populations rather than a fibroblast population. Current studies more often support a cooperative relationship between SPP1⁺ macrophages and CAF populations, particularly FAP⁺ fibroblasts, in tumor progression, stromal or desmoplastic remodeling, immune exclusion, and poor clinical outcome. In colorectal cancer, single-cell and spatial analyses demonstrated a strong positive association and close spatial interaction between FAP⁺ fibroblasts and SPP1⁺ macrophages, with co-enrichment of these two populations associated with worse progression-free survival [71]. Similar findings have also been reported in NSCLC, where SPP1⁺ macrophages and FAP⁺ fibroblasts are enriched together and tend to localize in close proximity, and their co-enrichment is associated with poorer overall survival [72]. Because CAF_7 in our model arises from TAMs via MMT, retention of SPP1 is biologically plausible. In this context, shared SPP1 expression may suggest a biological link between CAF_7 and SPP1⁺ macrophage states, although the precise relationship between these states remains to be clarified.

By illuminating this novel macrophage–CAF axis, our study fills a critical gap in the metastatic TME paradigm and suggests new therapeutic approaches. While CAFs are known to contribute to immunotherapy resistance, most anti-CAF strategies have treated fibroblasts as a homogeneous target [14, 73–75]. Our identification of a specific, immunosuppressive CAF subset arising from macrophages introduces a more precise target for intervention. Biologically, these results add a new dimension to the “seed and soil” model of metastasis: not only do cancer “seeds” exploit the pre-existing “soil” of bone [76], but they actively transform the soil by reprogramming immune cells into stromal facilitators. From a therapeutic standpoint, the discovery of ASCL2 as an actionable node in this process is particularly encouraging. Transcription factors can be challenging to inhibit directly, but our proof-of-concept experiments used macrophage-targeted ASCL2 knockdown to effectively block MMT and its downstream consequences. Strikingly, ASCL2 silencing in TAMs reversed the immunosuppressive phenotype–reducing Treg accumulation and restoring functionality of exhausted CD8⁺ T cells–and thereby suppressed metastatic tumor growth. These outcomes suggest that targeting ASCL2 or its upstream activators could reprogram the metastatic microenvironment from immunosuppressive to immuno-permissive. In practical terms, therapeutic strategies such as nanoparticle-delivered siRNA against ASCL2 or small-molecule inhibitors of pathways that induce ASCL2 (e.g., Wnt) could be explored. Importantly, our data show that ASCL2-targeted therapy can synergize with immune checkpoint blockade: in our models, PD-1 inhibition alone had minimal effect on bone lesions, but combining it with ASCL2 knockdown produced robust tumor regression and extended survival. This synergistic effect underscores the potential clinical benefit of simultaneously attacking the tumor’s stromal defenses and immune checkpoints. Patients with bone metastases–who often respond poorly to ICIs [5, 36]–might particularly benefit from such combination strategies to overcome the formidable immune barriers in the bone milieu. Notably, the ASCL2–Il6ra/IL6R connection also suggests a pragmatic translational angle, given that IL-6/IL6R-targeting agents and readily measurable downstream readouts are well established in other clinical settings [77], thereby providing an actionable rationale for exploring combination strategies.

Despite the insights gained, our study has limitations that warrant consideration. First, the sample size for bone-metastasis tissues was limited; although we integrated data across multiple patients and stages, larger patient cohorts will be needed to validate the generality of the seven CAF subsets and the prognostic value of CAF_7. Second, our in vivo functional and therapeutic evaluations were performed in an intratibial setting that models established intraosseous lesions; therefore, it does not address whether targeting the ASCL2–MMT axis can additionally influence bone homing/colonization efficiency during the seeding stage. Future studies using systemic bone-metastasis models (e.g., intracardiac or intra-arterial injection) will be important to directly test seeding-stage homing/colonization effects. Finally, the therapeutic strategies demonstrated (peritumoral siRNA delivery and combination with PD-1 blockade), while effective in our preclinical setting, require careful evaluation for safety, delivery, and efficacy in humans. Off-target effects of ASCL2 inhibition and potential compensatory mechanisms (e.g., other immunosuppressive cells in bone) are important considerations before clinical translation.

Looking ahead, several questions merit investigation. An immediate priority is to elucidate what upstream signals in the bone metastatic milieu drive ASCL2 in TAMs. Dissecting the bone secretome and signaling pathways in metastasis-bearing bones could identify druggable factors (such as Wnt ligands or TGF-β family members) that induce the ASCL2–MMT program. In addition, clarifying how ASCL2 couples to IL-6/IL6R signaling in vivo—particularly whether macrophage Il6ra/IL6R primarily reinforces MMT in an autocrine manner or acts through paracrine trans-signaling to shape T cell dysfunction—will help refine mechanistic and therapeutic hypotheses. In parallel, developing macrophage-specific delivery systems (e.g., lipid nanoparticles or engineered exosomes) for ASCL2 inhibitors or RNAi would enhance the translational feasibility of targeting this pathway. Given the tractability of IL-6/IL6R pathway modulation, it will also be informative to test whether IL-6/IL6R blockade phenocopies or potentiates ASCL2-directed reprogramming in bone lesions. It is also worth examining whether MMT occurs in other metastatic sites (such as liver or brain) and whether similar regulatory axes are involved there, which could broaden the impact of our findings. A logical next step will be preclinical trials combining an ASCL2-targeting strategy with immune checkpoint inhibitors in rigorous bone metastasis models, to formally test the synergistic efficacy suggested by our results. Such studies will determine whether reprogramming TAMs can consistently boost anti-tumor immunity and improve immunotherapy responses in metastatic disease. In sum, further investigation along these lines will not only refine our understanding of metastatic niche biology but also pave the way for innovative immunostromal therapies.

Collectively, our work establishes a mechanistic link between tumor-associated macrophages and immunosuppressive CAFs in the context of lung cancer bone metastasis. By demonstrating that ASCL2-driven MMT enables metastases to create a self-sustaining immunosuppressive niche, we provide new insights into how metastatic tumors evade immunity. These findings highlight a promising therapeutic target—the macrophage-to-CAF conversion process–and suggest that intervening at this nexus, especially in combination with immune checkpoint blockade, could substantially improve outcomes for patients with metastatic cancer. Our study thus contributes to a growing framework for reprogramming the metastatic microenvironment to thwart immune evasion and enhance immunotherapy efficacy.

## Conclusion

In summary, integrating single-cell transcriptomes from NSCLC primary tumors and bone metastases delineates seven CAF states and uncovers a bone-enriched subset, CAF_7, that progressively expands with disease advancement. CAF_7 exhibits a hybrid program combining macrophage-lineage and MHC-II traces with myofibroblast/ECM features, and convergent trajectory and perturbation evidence supports its emergence through macrophage-to-myofibroblast transition (MMT). In established bone lesions, higher CAF_7 abundance is associated with a profoundly immunosuppressive milieu marked by Treg enrichment and CD8⁺ T-cell exhaustion. Mechanistically, ASCL2 is identified as a central regulator of this transition in the bone metastatic context; macrophage-intrinsic ASCL2 silencing suppresses CAF_7-like formation, alleviates Treg expansion and CD8⁺ T-cell dysfunction, and limits bone metastatic tumor progression in the intratibial setting. We further connect ASCL2 to an Il6ra/IL6R effector axis via promoter occupancy and transcriptional activation, and show that macrophage Il6ra knockdown recapitulates key immunostromal and tumor-control phenotypes. Notably, ASCL2 targeting cooperates with PD-1 blockade to achieve superior tumor control and prolonged survival in preclinical models. Collectively, these findings highlight macrophage plasticity as a previously underappreciated source of immunosuppressive CAFs in NSCLC bone metastases and provide a mechanistic rationale for therapeutically reprogramming the metastatic niche by disrupting the ASCL2-driven MMT pathway.

## Supplementary Information

Supplementary Material 1.

## Data Availability

The data that support the findings of this study are available from the corresponding author upon reasonable request.
