# Supplementary material for: ASCL2-mediated macrophage-myofibroblast transition generates immunosuppressive CAF_7 in NSCLC bone metastases
Source: J Exp Clin Cancer Res. 2026 May 18;45:148. doi: 10.1186/s13046-026-03735-1 (PMC13325787; doi:10.1186/s13046-026-03735-1)
Supplement: Supplementary file 1 — Supplementary Material 1. [file 13046_2026_3735_MOESM1_ESM.docx]

Supporting Information

ASCL2-Mediated Macrophage-Myofibroblast Transition Generates Immunosuppressive CAF_7 in NSCLC Bone Metastases

Jinfeng Wang^1,2†^, Jin Qian^1,2†^, Xiujia Yang^3†^, Chongquan Huang^1,2†^, Tiantian Wei^1,2^, Jielong Zhou^1,2^, Guoqing Zhong^8*^, Zhenhai Zhang^4,5,6,7*^, Yu Zhang^1,2*^

**Figure S1. Distinct transcriptional and prognostic characteristics of CAF_7 in bone metastases versus advanced-stage primary NSCLC.**
(A) Venn diagram comparing the top 1,000 highly expressed genes in CAF_7 derived from BM and advanced_LUNG samples. Only 360 genes (22%) were shared, suggesting divergent transcriptional profiles.
(B) GO Biological Process (GOBP) enrichment of BM- and advanced_LUNG-specific CAF_7 gene signatures. Functional overlap was minimal, with only 6.09% (48/788) shared terms, indicating context-specific CAF_7 reprogramming during bone metastasis.
(C) Kaplan–Meier survival analysis using a CAF_7 signature derived from advanced-stage primary NSCLC tumors (advanced_LUNG). CIBERSORTx deconvolution of bulk transcriptomic data from six TCGA cancer cohorts revealed significant prognostic associations in only two cancer types, indicating limited and less consistent predictive value compared to bone metastasis–derived CAF_7. These findings underscore the enhanced prognostic relevance of CAF_7 populations reprogrammed in the bone metastatic microenvironment.

**Figure S2. Additional analyses supporting coordinated ECM/myofibroblast features of CAF_7 and complementary in vitro and in vivo evidence for macrophage-to-CAF_7-like remodeling.**
(A) Single-cell heatmap of representative ECM/myofibroblast genes in Macro, Other_CAF, and CAF_7 from the bone-metastasis scRNA-seq dataset. Columns represent individual cells and rows represent genes; expression is shown as scaled values. Macro cells show sparse and patchy expression of ECM/myofibroblast-related genes, whereas Other_CAF and CAF_7 show broad within-cell co-expression of multiple ECM/myofibroblast genes.
(B) Number of detected ECM/myofibroblast genes per cell in Macro, Other_CAF, and CAF_7, based on the representative ECM/myofibroblast gene set shown in (A). Compared with Macro, both Other_CAF and CAF_7 show markedly higher per-cell ECM/myofibroblast gene detection.
(C) Fraction of cells meeting increasingly stringent thresholds for coordinated ECM/myofibroblast gene detection (>10, >15, or >20 detected ECM/myofibroblast genes per cell) in Macro, Other_CAF, and CAF_7. Other_CAF and CAF_7 maintain high fractions of cells across all thresholds, whereas Macro remains low.
(D) Relationship between the number of detected ECM/myofibroblast genes per cell and the ECM/myofibroblast module score in Macro, Other_CAF, and CAF_7. Each point represents a single cell. Fitted trend lines show that module scores increase as more ECM/myofibroblast genes are detected, with stronger upward trends in Other_CAF and CAF_7 than in Macro.
(E) Paired comparison of Phagolysosome, lipid handling/cholesterol efflux, and ECM/Myofibroblast module scores between Macro and CAF_7 within the same bone-metastasis samples. The Phagolysosome module was defined by LAMP1, LAMP2, RAB7A, CTSD, and CTSB, and the lipid handling/cholesterol efflux module was defined by ABCA1, ABCG1, APOE, LIPA, PLIN2, FABP5, PPARG, and NR1H3. Sample-level scores were calculated as the mean module score of each cell type within each sample. Only samples with at least 5 Macro cells and 5 CAF_7 cells were included. Compared with Macro, CAF_7 showed lower Phagolysosome and lipid handling/cholesterol efflux scores but a higher ECM/Myofibroblast score (p = 0.008, p = 0.016, and p = 0.008, respectively).
(F) qPCR analysis of BMDMs cultured with or without LLC-CM. LLC-CM decreases macrophage-lineage/antigen-presentation–associated transcripts (F4/80, CD68, and CD74) and increases myofibroblast/CAF-related transcripts (FAP, α-SMA/ACTA2, and VEGF) as well as immunoregulatory/secretome-related transcripts (MRC1, HAVCR2, and SPP1) (n = 3; *p < 0.05, **p < 0.01, ***p < 0.001, ****p < 0.0001).
(G) Representative immunofluorescence images and quantitative analysis demonstrate that macrophage depletion with clodronate liposomes significantly reduces both CD68⁺α-SMA⁺ CAF_7-like cells and the overall α-SMA⁺ stromal compartment in murine lung cancer bone metastases (n = 4, ***p < 0.001, ****p < 0.0001). Scale bars, 20 μm. Colors: CD68 red; α-SMA green; DAPI blue. α-SMA indicates myofibroblast/activated CAF features and CD68 reflects a macrophage-lineage imprint; CD68⁺α-SMA⁺ cells are used as an operational readout of CAF_7-like cells.
(H) Flow-cytometric gating strategy for isolation of viable tdTomato⁺FAP⁺ and tdTomato⁺FAP⁻ populations from dissociated bone metastatic lesions of LysM-Cre;Rosa26-tdTomato mice. Sequential gating was applied to exclude debris, select singlets, identify viable DAPI-negative cells, enrich tdTomato⁺ cells, and separate tdTomato⁺FAP⁺ and tdTomato⁺FAP⁻ fractions.

**Figure S3. Donor-tracing and downstream analyses support acquisition of CAF_7-like features by GFP-labeled BMDMs in bone metastatic lesions.**
(A) Immunofluorescence analysis of bone metastatic lesions from the macrophage depletion/reconstitution intratibial model. Endogenous macrophages were depleted with clodronate liposomes, and GFP-labeled BMDMs were co-injected with LLC cells into the tibia at a 1:1 ratio. Representative images show GFP-labeled BMDMs (green), α-SMA (red), and DAPI (blue), with merged images indicating co-expression of GFP and α-SMA.
(B) Flow-cytometric gating strategy for isolation of viable GFP⁺FAP⁺ and GFP⁺FAP⁻ populations from dissociated bone metastatic lesions. Sequential gating was applied to exclude debris, select singlets, identify viable DAPI-negative cells, enrich GFP⁺ cells, and separate GFP⁺FAP⁺ and GFP⁺FAP⁻ fractions.
(C) qPCR analysis of sorted GFP⁺FAP⁺ and GFP⁺FAP⁻ populations isolated from bone lesions. Compared with the GFP⁺FAP⁻ fraction, GFP⁺FAP⁺ cells showed increased expression of FAP and α-SMA together with reduced expression of F4/80 and CD68 (n = 3; *p < 0.05, **p < 0.01, ***p < 0.001, ****p < 0.0001).
(D) Gene set enrichment analysis (GSEA) of the ranked differential-expression gene list derived from donor-derived GFP⁺FAP⁺ and GFP⁺FAP⁻ populations. Representative enrichment plots show that GFP⁺FAP⁺ cells are enriched for pathways associated with extracellular matrix organization and myofibroblast-associated contractile function. NES, normalized enrichment score; Adj.P, adjusted P value.

**Figure S4. Cell–cell communication landscape of CAF_7 in lung cancer bone metastases.**
(A) CellChat interaction network plots showing the number (left) and strength (right) of outgoing signals from CAF_7 to various immune and stromal cell populations. Notably, CAF_7 displays extensive interactions with CD4⁺ Tregs, CD4⁺ T, CD8⁺ T, and CD8⁺ Tex subsets.

**Figure S5. ASCL2 regulon features and ASCL2 expression correlate with CAF programs and adverse prognosis across cancers.**
(A) In TGF-β1–induced BMDMs and murine intratibial bone-metastasis lesions (BM) versus sham, a qPCR pre-screen of SCENIC-nominated TFs shows ASCL2 as the most strongly induced factor and consistently upregulated across both settings, whereas LYL1, ZNF467, EGR2, and ETV5 change modestly or inconsistently; representative bar plots and quantifications are shown (in vitro n = 3; in vivo n = 3, *p < 0.05, **p < 0.01, ***p < 0.001, ****p < 0.0001).
(B) Transcriptional regulatory network analysis using pySCENIC identified key ASCL2-associated regulon targets, including SASH3, IL6R, and FCGR2B.
(C) Pan-cancer analysis (TCGA) revealed a positive correlation between ASCL2-TF module scores and CAF signatures (R = 0.21, p < 2.2e-16).
(D) Kaplan–Meier survival analyses revealed that elevated ASCL2 expression predicts unfavorable prognosis across multiple cancer types, including lung adenocarcinoma (LUAD), lung squamous carcinoma (LUSC), hepatocellular carcinoma (LIHC), breast cancer (BRCA), kidney renal clear cell carcinoma (KIRC), esophageal squamous cell carcinoma (ESCA), testicular germ cell tumor (TGCT), thymoma (THYM).

**Figure S6. ASCL2 knockdown attenuates TGF-β1–induced CAF-like morphology, and ASCL2^res rescue reverses siASCL2-driven marker changes during MMT in BMDMs.**
(A) Representative bright-field images of BMDMs show that TGF-β1 induces a CAF-like morphology in siNC-BMDMs, whereas ASCL2 knockdown (siASCL2-BMDMs) attenuates this morphological change, consistent with the immunofluorescence results in Figure 5A,B.
(B) Western blot with densitometric quantification of BMDMs under TGF-β1–induced MMT conditions transfected with siNC+EV (negative-control siRNA + empty vector), siASCL2+EV, or siASCL2+ASCL2^res (RNAi-resistant ASCL2 rescue; labeled “A2-res”). The blots confirm ASCL2 rescue and show associated changes in IL6R, macrophage-lineage markers (F4/80, CD68, CD74), CAF markers (FAP, α-SMA, VEGF), and immunoregulatory proteins (MRC1, HAVCR2). Compared with siNC+EV, siA2+EV reduced ASCL2 and IL6R and decreased FAP, α-SMA, VEGF, MRC1, and HAVCR2, while increasing F4/80, CD68, and CD74; A2-res largely reversed these marker changes toward siNC+EV (n = 3; *p < 0.05, **p < 0.01, ***p < 0.001, ****p < 0.0001).

**Figure S7. Flow cytometry gating strategies for MMT, Treg differentiation, and CD8⁺ T-cell exhaustion analyses.**
(A) Representative plots illustrate the flow cytometry gating sequence used to identify macrophage-to-myofibroblast transition (MMT) in vitro. Debris and doublets were excluded, and viable single cells were gated by DAPI negativity. Cells positive for α-SMA expression were considered MMT-like.
(B) Gating strategy for identifying MMT in vivo from mouse bone metastasis lesions. MMT was defined as α-SMA⁺CD68⁺ cells; within this gate, ASCL2⁺ events were quantified to define the ASCL2⁺ MMT subset (α-SMA⁺CD68⁺ASCL2⁺).
(C) Representative gating strategy for defining regulatory T cells (Tregs) in the in vitro co-culture assay. Following live CD45⁺CD3⁺CD4⁺ gating, Tregs were identified as CD25⁺FOXP3⁺ cells. In vivo samples were analyzed using the same sequential gating strategy.
(D) Representative gating strategy for detecting exhausted CD8⁺ T cells in the in vitro co-culture assay. After gating on live CD45⁺CD3⁺CD8⁺ T cells, co-expression of PD-1 and HAVCR2 (TIM-3) was used to quantify exhausted CD8⁺ T cells. In vivo samples were analyzed using the same sequential gating strategy.

**Table S1. The clinical characteristics of patients used for single-cell RNA sequencing**

| Sample ID | Patient ID | Disease | Age | Sex | Smoking Status | Stage | Metastasis Site | EGFR | PD-L1 (%) |
| --- | --- | --- | --- | --- | --- | --- | --- | --- | --- |
| NEURO0 | P01 | LUAD | 52.0 | Female | No | IV | Pelvis | unknown | unknown |
| NEURO1 | P02 | LUAD | 73.0 | Female | No | IV | Rib | unknown | unknown |
| NEURO2 | P03 | LUAD | 66.0 | Female | No | IV | Spine | unknown | <1 |
| NEURO3 | P04 | LUAD | 72.0 | Male | No | IV | Spine | unknown | <1 |
| NEURO4 | P05 | LUAD | 64.0 | Male | No | IV | Spine | unknown | unknown |
| NEURO5 | P06 | LUAD | 70.0 | Male | No | IV | Femur | unknown | unknown |
| NEURO6 | P07 | ASC | 68.0 | Female | No | IV | Humerus | unknown | 100 |
| NEURO7 | P08 | ASC | 55.0 | Male | No | IV | Femur | unknown | ≈20 |
| NEURO8 | P09 | LUAD | 70.0 | Female | No | IV | Spine | unknown | 20 |
| NEURO9 | P10 | LUAD | 71.0 | Male | Yes | IV | Spine | unknown | ≈60 |

Note: LUAD, lung adenocarcinoma; ASC, adenosquamous carcinoma.

**Table S2. siRNA sequences used in this study**

| siRNA Name | Sense (5′–3′) | Antisense (5′–3′) |
| --- | --- | --- |
| siASCL2 | CCGCGUAAAGCUGGUAAACUU(dT)(dT) | AAGUUUACCAGCUUUACGCGG(dT)(dT) |
| siIl6ra | GACUAUUUAUGCUCCCUGAAUTT | AUUCAGGGAGCAUAAAUAGUCTT |
| siNC | UUCUCCGAACGUGUCACGUTT | ACGUGACACGUUCGGAGAATT |

**Table S3. Primer sequences used for qPCR**

| Target | Forward primer (5′ to 3′) | Reverse primer (5′ to 3′) |
| --- | --- | --- |
| LYL1 | AAAACTGAGATGGTATGTGCCTC | TGTCCCAGGTTTATCACTGGC |
| ZNF467 | GTCAGTCCCGGAGTCTTTCG | CACAGACCCTGGCGGG |
| ASCL2 | AAGCACACCTTGACTGGTACG | AAGTGGACGTTTGCACCTTCA |
| EGR2 | GCCAAGGCCGTAGACAAAATC | CCACTCCGTTCATCTGGTCA |
| ETV5 | GGCGACCTTTGATTGACAGAA | CCAGCTTCGTCAGACAGAGAG |
| F4/80 | CCCCAGTGTCCTTACAGAGTG | GTGCCCAGAGTGGATGTCT |
| CD68 | GGACCCACAACTGTCACTCAT | AAGCCCCACTTTAGCTTTACC |
| CD74 | CCGCCTAGACAAGCTGACC | ACAGGTTTGGCAGATTTCGGA |
| MRC1 | CTCTGTTCAGCTATTGGACGC | CGGAATTTCTGGGATTCAGCTTC |
| HAVCR2 | TCAGGTCTTACCCTCAACTGTG | GGGCAGATAGGCATTTTTACCA |
| SPP1 | AGCAAGAAACTCTTCCAAGCAA | GTGAGATTCGTCAGATTCATCCG |
| FAP | GTCACCTGATCGGCAATTTGT | CCCCATTCTGAAGGTCGTAGAT |
| α-SMA | CCCAACTGGGACCACATGG | TACATGCGGGGGACATTGAAG |
| Col1a1 | GCTCCTCTTAGGGGCCACT | CCACGTCTCACCATTGGGG |
| VEGF | GCACATAGAGAGAATGAGCTTCC | CTCCGCTCTGAACAAGGCT |
| GAPDH | CCTGGAGAAACCTGCCAAGTATG | AGAGTGGGAGTTGCTGTTGAAGTC |

**Table S4. Candidate primer sequences for Il6ra ChIP–qPCR**

| Target (ChIP–qPCR) | Sense (5′–3′) | Antisense (5′–3′) |
| --- | --- | --- |
| Il6ra (ChIP–qPCR amplicon, candidate regulatory region) | CGTTGCCTCTGCAGTAGCCTTCAA | TCTGCTTCGAGCCCAGAGATTGTG |
| Il6ra (ChIP–qPCR amplicon, candidate regulatory region) | CTGCAGGAAGTAACCCCCTC | CACATGCACGTGACACACAG |
| Il6ra (ChIP–qPCR amplicon, candidate regulatory region) | GACTCAAAAGCGCACACTGG | GCTTCGAGCCCAGAGATTGT |
| Il6ra (ChIP–qPCR amplicon, candidate regulatory region) | TAACCGCTGAGCCATCTCAC | CAGACTCCACCAAGGTTCCC |
| Il6ra (ChIP–qPCR amplicon, candidate regulatory region) | TGAGCTACCATGTTGCTGGG | CTCCACCAAGGTTCCCTTCC |

**Table S5. Antibodies used in this study**

| Antibody Name | Company | Catalog Number |
| --- | --- | --- |
| PerCP/Cyanine5.5 anti-mouse CD45 Antibody | Biolegend | Cat#157208 |
| PE/Cyanine7 anti-mouse CD3 Antibody | Biolegend | Cat#100220 |
| PE anti-mouse CD4 Antibody | Biolegend | Cat#100408 |
| FITC anti-mouse CD25 Antibody | Biolegend | Cat#101908 |
| Alexa Fluor 647 anti-mouse FOXP3 Antibody | Biolegend | Cat#126408 |
| PerCP/Cyanine5.5 anti-mouse CD8a Antibody | Biolegend | Cat#100734 |
| PE anti-mouse CD279 (PD-1) Antibody | Biolegend | Cat#135206 |
| APC anti-mouse CD366 (Tim-3) Antibody | Biolegend | Cat#134008 |
| PE anti-mouse FAP Antibody | Novus Biologicals | Cat#FAB9727P |
| FITC anti-mouse FAP Antibody | Novus Biologicals | Cat#FAB9727F |
| CD68 Antibody | Affinity Biosciences | Cat#DF7518 |
| ASCL2 Antibody | Bioss | Cat#bs-12349R |
| TCF-1 Antibody | CST | Cat#2203S |
| IL6R | Proteintech | Cat#23457-1-AP |
| HAVCR2 Antibody | Affinity Biosciences | Cat#DF6979 |
| Alpha-SMA Antibody | Affinity Biosciences | Cat#AF1032 |
| GAPDH Antibody | Affinity Biosciences | Cat#AF7021 |
| CD74 Chimeric Recombinant Rabbit Monoclonal Antibody | Invitrogen | Cat#MA5-48279 |
| F4/80 Antibody | Affinity Biosciences | Cat#DF2789 |
| FAP Antibody | Affinity Biosciences | Cat#AF5344 |
| VEGFA Antibody | Affinity Biosciences | Cat#AF5131 |
| CD4 Antibody | Affinity Biosciences | Cat#DF16080 |
| FOXP3 Antibody | Affinity Biosciences | Cat#AF6544 |
| Anti-CD8 alpha antibody | Abcam | Cat#ab316778 |
| Anti-PD1 Antibody | Abcam | Cat#ab309363 |
| Alexa Fluor 488 AffiniPure Donkey Anti-Mouse IgG (H+L) | Jackson ImmunoResearch | Code: 715-545-151 |
| Cy3 AffiniPure Donkey Anti-Mouse IgG (H+L) | Jackson ImmunoResearch | Code: 715-165-150 |
| Alexa Fluor 488 AffiniPure Donkey Anti-Rabbit IgG (H+L) | Jackson ImmunoResearch | Code: 711-545-152 |
| Cy3 AffiniPure Donkey Anti-Rabbit IgG (H+L) | Jackson ImmunoResearch | Code: 712-165-152 |
| Horseradish enzyme labeled goat anti-rabbit IgG (H+L) | ZSGB-Bio | Cat#ZB-2301 |
| Horseradish enzyme labeled goat anti-mouse IgG (H+L) | ZSGB-Bio | Cat#ZB-2305 |
| Goat anti-rabbit IgG, Alexa Fluor 532 | Bioss | Cat#11072 |
| Goat anti-rabbit IgG, Alexa Fluor 488 | Abcam | Cat#ab150077 |
| Goat anti-rabbit IgG, Alexa Fluor cy3 | Abcam | Cat#ab150078 |
| Goat anti-rabbit IgG, HRP / TSA-430 system | Abcam / Wuhan Ruiseqi Biotech | Cat#ab205718 / TSA-05 |
| Goat anti-rabbit IgG, Alexa Fluor cy5 | Abcam | Cat#ab6564 |

Note: Antibodies used for immunofluorescence, flow cytometry, and Western blot are included.

**Table S6. Signature gene sets used for CAF_7, Treg, and exhausted T cell scoring analyses**

| **Signature** | **Genes** |
| --- | --- |
| CAF_7 | CD68, CD163, MRC1, CD14, MS4A7, AIF1, MSR1, FCGR2A, FCGR3A, TYROBP, C1QA, C1QB, CSF1R, CYBB, CLEC7A, HLA-DRA, HLA-DRB1, HLA-DPA1, HLA-DPB1, HLA-DQA1, HLA-DQB1, HLA-DMA, CD74, CIITA, IFI30, SPI1, IRF8, CCL2, CCL3, CCL4, CCL3L1, CXCL2, CXCL3, CXCL8, CXCL16, IL1B, IL18, TNFSF13B, SPP1, HAVCR2, VSIR, LGALS9, TNFRSF1B |
| Treg | FOXP3, IL2RA, CTLA4, TIGIT, CCR8, ENTPD1, IL10 |
| Exhausted | PDCD1, TIGIT, LAG3, HAVCR2, TOX |
